# High production of triterpenoids in *Yarrowia lipolytica* through manipulation of lipid components

**DOI:** 10.1186/s13068-020-01773-1

**Published:** 2020-07-29

**Authors:** Jin-Lai Zhang, Qiu-Yan Bai, Yang-Zi Peng, Jie Fan, Cong-Cong Jin, Ying-Xiu Cao, Ying-Jin Yuan

**Affiliations:** 1 Frontier Science Center for Synthetic Biology and Key Laboratory of Systems Bioengineering (Ministry of Education), School of Chemical Engineering and Technology, Tianjin University, Tianjin, 300072 China; 2 Collaborative Innovation Center of Chemical Science and Engineering (Tianjin), Tianjin University, Tianjin, 300072 China

**Keywords:** Triterpenoids, Lipid manipulation, Unsaturated fatty acids, Cell morphology, *Yarrowia lipolytica*

## Abstract

**Background:**

Lupeol exhibits novel physiological and pharmacological activities, such as anticancer and immunity-enhancing activities. However, cytotoxicity remains a challenge for triterpenoid overproduction in microbial cell factories. As lipophilic and relatively small molecular compounds, triterpenes are generally secreted into the extracellular space. The effect of increasing triterpene efflux on the synthesis capacity remains unknown.

**Results:**

In this study, we developed a strategy to enhance triterpene efflux through manipulation of lipid components in *Y. lipolytica* by overexpressing the enzyme Δ9-fatty acid desaturase (*OLE1*) and disturbing phosphatidic acid phosphatase (*PAH1*) and diacylglycerol kinase (*DGK1*). By this strategy combined with two-phase fermentation, the highest lupeol production reported to date was achieved, where the titer in the organic phase reached 381.67 mg/L and the total production was 411.72 mg/L in shake flasks, exhibiting a 33.20-fold improvement over the initial strain. Lipid manipulation led to a twofold increase in the unsaturated fatty acid (UFA) content, up to 61–73%, and an exceptionally elongated cell morphology, which might have been caused by enhanced membrane phospholipid biosynthesis flux. Both phenotypes accelerated the export of toxic products to the extracellular space and ultimately stimulated the capacity for triterpenoid synthesis, which was proven by the 5.11-fold higher ratio of extra/intracellular lupeol concentrations, 2.79-fold higher biomass accumulation and 2.56-fold higher lupeol productivity per unit OD in the modified strains. This strategy was also highly efficient for the biosynthesis of other triterpenes and sesquiterpenes, including α-amyrin, β-amyrin, longifolene, longipinene and longicyclene.

**Conclusions:**

In conclusion, we successfully created a high-yield lupeol-producing strain via lipid manipulation. We demonstrated that the enhancement of lupeol efflux and synthesis capacity was induced by the increased UFA content and elongated cell morphology. Our study provides a novel strategy to promote the biosynthesis of valuable but toxic products in microbial cell factories.

## Background

Triterpenoids are a class of antineoplastic natural products that serve as significant resources of pharmaceuticals for the treatment of various diseases, exhibiting properties such as anti-inflammatory, antibacterial, antifungal, antioxidation, anti-HIV, and immunity-enhancing properties [1]. To keep pace with the increasing commercial demand for triterpenoids, metabolic engineering of cell factories has emerged as a promising and attractive alternative in industrial production and is an environmentally friendly and cost-efficient approach that is independent of plants [2].

Nevertheless, terpenoids, known for their in vivo antifungal activity, are generally highly toxic to microorganisms, inducing apoptosis and having a negative impact on terpene production [3–8]. For instance, lupeol, a typical triterpenoid, caused severe damage to cell viability even at a relatively low concentration of 60 mg/L [3, 8]. To decrease the intractable cytotoxicity, extensive efforts aimed at creating oleaginous subcellular organelles by altering lipid-droplet composition and size potentially improved the terpene partition coefficient in oil droplets and the storage space so that lipophilic terpenes can accumulate in these compartments [9–15]. For instance, researchers successfully increased lycopene accumulation by creating supersized lipid droplets through manipulation of triacylglycerol (TAG) metabolism in *Saccharomyces cerevisiae*, which resulted in the highest yield (73.3 mg/g cdw and 2.37 g/L lycopene) reported in *S. cerevisiae* to date [10]. Similarly, knocking out *POX1* to *POX6* and *GUT2* in *Yarrowia lipolytica* increased the size of lipid bodies, which enabled *Y. lipolytica* to withstand higher lycopene concentrations [11]. Gao et al. [12] successfully applied lipid droplets as a storage sink to enhance β-carotene production in *Y. lipolytica*, resulting in a β-carotene yield of up to 4 g/L in fed-batch fermentation, the highest level attained so far.

Notably, the above strategies were biased toward the biosynthesis of tetraterpenoids with large molecules, which can accumulate in lipophilic compartments. However, terpenoids with relatively small molecules, such as triterpenes and flavonoids, are generally excreted into the extracellular space [16–18]. These products preferentially cross the plasma membrane via passive diffusion since the cell membrane is mainly composed of a lipid bimolecular layer [19]. As such, two-phase fermentation is widely utilized to extract terpenes from cells into extracellular lipophilic solvent and thus reduce product cytotoxicity [6]. However, the relatively low permeability of the cell membrane is still a barrier to the efflux of triterpenoids [19]. Manipulation of the lipid composition in membranes, such as the unsaturated fatty acid/saturated fatty acid (UFA/SFA) ratio or the membrane phospholipid content, is an efficient approach to tune membrane permeability and potentially alter the extra/intracellular partition coefficient of terpenes [15, 19–26]. For example, upregulation of SFA content in *E. coli* resulted in membrane tightening with enhanced tolerance to toxic n-hexane in culture [25]. Besides, yeast cells can resist the damage caused by extracellular ethanol by altering membrane phospholipid properties [26]. However, these modifications are mostly adopted to reduce cell membrane permeability to block the entry of extracellular hazardous substances. Few applications are committed to promoting toxic terpene secretion through lipid regulation in microbial cell factories.

**Fig. 1:**
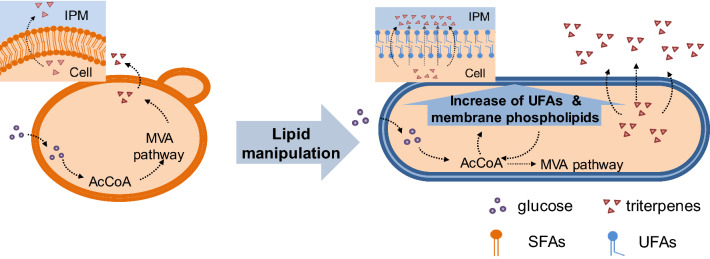
Schematic diagram of lipid metabolism engineering to improve triterpene production in *Y. lipolytica*. The strains without lipid manipulation possessed relatively high levels of saturated fatty acids and exhibited spherical cells (left). After regulating lipid metabolism, the unsaturated fatty acid content increased twofold, and an elongated cell morphology was obtained, facilitating lupeol efflux from the cell to the lipophilic solvent isopropyl myristate, thereby improving cell activity and the capacity for single-cell triterpene synthesis (right). AcCoA, acetyl coenzyme A; UFAs, unsaturated fatty acids; SFAs, saturated fatty acids; IPM, isopropyl myristate

As a “generally regarded as safe” (GRAS) host, *Y. lipolytica* has emerged as an attractive host preferentially employed in the production of numerous pharmaceuticals and nutraceuticals [27]. The naturally high flux of acetyl-CoA in *Y. lipolytica* provides abundant terpene synthesis precursors. Moreover, *Y. lipolytica* is a remarkable oleaginous yeast in which lipid accumulation accounts for over 40% of the dry cell weight (DCW) [28]. The tractability of lipid metabolism in *Y. lipolytica* makes it a promising platform organism for terpene biosynthesis [9]. However, the cytotoxicity of terpenes with antimicrobial activities and the resulting metabolic burdens have led to great challenges in the biomanufacturing of terpenes in *Y. lipolytica* [3–5, 8, 28]. Herein, we applied a strategy of engineering lipid components to achieve high production of lupeol, a pentacyclic triterpene, in *Y. lipolytica*. Specifically, we (i) established a heterologous lupeol synthesis pathway in *Y. lipolytica* by chromosomally integrating genes encoding lupeol synthases from different sources, (ii) optimized the precursor supply by engineering structural genes in the mevalonate (MVA) pathway, (iii) significantly elevated lupeol biosynthesis by disturbing essential genes in lipid metabolism, and (iv) revealed accelerated export of lupeol caused by a twofold increase in the UFA proportion and markedly elongated cell morphology (Fig. 1). After doing so, the titer of lupeol in the organic phase was 381.67 mg/L, and the total production reached 411.72 mg/L in shake flasks, representing the highest production reported to date. This strategy was also highly efficient for the biosynthesis of other triterpenes and sesquiterpenes, providing new insights for improving the production of valuable products in microbial cell factories.

## Results

### Establishment of lupeol synthesis in *Y. lipolytica*

Lupeol, a pentacyclic triterpene, is an important intermediate metabolite from the conversion of 2,3-oxidosqualene to a series of lupane-type triterpenoids and has attracted increasing attention due to its anti-HIV, anticancer and anti-inflammatory activities [3, 29, 30]. To produce lupeol from acetyl-CoA through the MVA pathway in *Y. lipolytica*, we assembled the following heterologous genes encoding lupeol synthases from different sources in strain ATCC 201249: *AtLus* from *Arabidopsis thaliana, GuLus* from *Glycyrrhiza uralensis*, *OeLus* from *Olea europaea*, *BgLus* from *Bruguiera gymnorhiza*, *KdLus* from *Kalanchoe daigremontiana* and *RcLus* from *Ricinus communis*. All of these genes were codon-optimized (Additional file 2: Table S1) and integrated into the *Ku70* site in the genome of *Y. lipolytica* under the control of the Hp4d promoter. Then, we quantified lupeol production after 5 days of cultivation. Of these variants, strain LU-6 containing *RcLus* achieved the highest lupeol titer of 12.40 mg/L (Additional file 1: Fig. S1A) and was designated the initial strain.

Subsequently, we sought to improve the expression of *RcLus* by testing other promoters [31–33] (Additional file 1: Fig. S1B) and changed the subcellular locations of lupeol synthase by fusing different subcellular localization signal peptides to *RcLus* (Additional file 2: Table S2), which was confirmed by laser scanning confocal microscopy analysis (Additional file 1: Fig. S1C). Collectively, the strain LU-9 containing the *RcLus* gene expressed by the pTEFin promoter and localized in the cytosol achieved the highest lupeol titer of 29.00 mg/L, a 2.33-fold increase compared with the initial strain LU-6 (Additional file 1: Fig. S1D). Thus, an optimized heterologous lupeol synthesis pathway was successfully established in *Y. lipolytica*. Strain LU-9 was selected for subsequent engineering and was designated the control strain in this study.

### Engineering of MVA and lipid metabolism to improve lupeol production

**Fig. 2:**
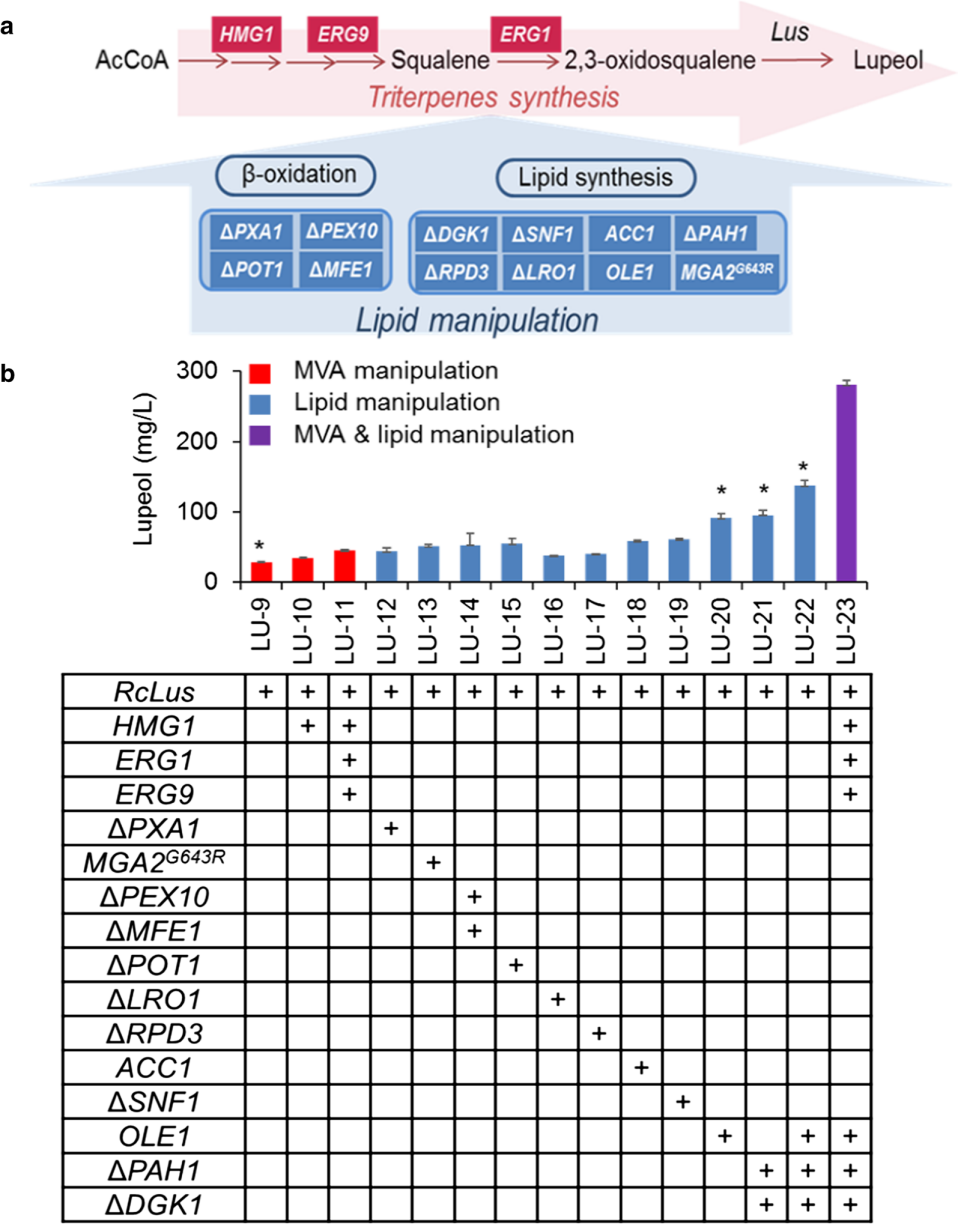
Effect of metabolic engineering of the MVA pathway and lipid metabolism on lupeol production. **a** Related modified genes involved in the MVA biosynthesis pathway and lipid manipulation are represented by red rectangles and blue rectangles, respectively. **b** Lupeol production by engineered strains. The strains selected for further investigations are marked with asterisks. Error bars represent ± SD of biological triplicates

To improve lupeol production, we first upregulated the MVA pathway by overexpressing the rate-limiting enzyme *HMG1* (3-hydroxy-3-methylglutaryl coenzyme A reductase) [34, 35] in strain LU-9, generating strain LU-10. It has been reported that synchronous overexpression of *ERG1* (squalene monooxygenase) and *ERG9* (squalene synthase) [36] exhibited cooperativity toward enhancing terpene synthesis [37]. Hence, we further used this combination based on *HMG1* overexpression to generate strain LU-11. In summary, *HMG1* and *ERG9* were expressed by the pTEFin promoter, and *ERG1* was controlled by the pEXP1 promoter, which was chromosomally integrated into the rDNA site of *Y. lipolytica,* marked with red rectangles in Fig. 2a. Compared with the control strain LU-9, the resulting strain LU-10 with single *HMG1* overexpression and LU-11 with *HMG1*, *ERG9* and *ERG1* overexpression presented only slight improvement of lupeol yield at 1.21- and 1.56-fold, respectively (Fig. 2b). The achieved yield of lupeol in the present host was still at a low level, which is consistent with the effect on amorphadiene biosynthesis in *Y. lipolytica* [35]. These results revealed that MVA pathway modifications could not be the main factor that limited terpene overproduction in microorganisms; instead, the real rate-limiting step might be the downstream process, such as terpene synthesis, storage or transport.

Lipid metabolism was proven to be an effective strategy that can relieve the downstream bottleneck of terpene synthesis, including by extending the storage capacity of lipophilic products or improving terpene synthase efficiency, thereby promoting terpene overproduction [9–13]. As such, we manipulated eight structural and regulatory genes related to lipid metabolism in LU-9 (Fig. 2a), including β-oxidation disruption (by knocking out *PXA1, MFE1, PEX10* or *POT1*) [38–40] and lipid synthesis regulation (by overexpressing the limiting step genes *ACC1, OLE1* and its activator *MGA2*^*G643R*^ and disturbing the regulatory and structural genes *RPD3, SNF1, LRO1, PAH1* and *DGK1*) [41, 42] (Fig. 2a and Additional file 1: Fig. S2). Overexpression of each gene was performed by homologous recombination, and knockout of each gene was achieved by the CRISPR/Cas9 system, generating a series of strains from LU-12 to LU-21 based on LU-9. Among these strains, LU-20, with overexpression of *OLE1*, and LU-21, with knockout of *PAH1*-*DGK1*, promoted lupeol production most significantly, showing 3.16- and 3.30-fold improvement over strain LU-9, respectively. Then, a strain of LU-22 was generated by combination of overexpression of *OLE1* and knockout of *PAH1*-*DGK1*, which further improved lupeol production to 137.52 mg/L, 4.74-fold higher than the value for the strain LU-9.

Subsequently, manipulation of the MVA pathway and lipid metabolism were combined, generating strain LU-23 (with overexpression of *RcLus*, *HMG1, ERG1, ERG9*, and *OLE1* and knockout of *PAH1*-*DGK1*). As shown in Fig. 2b, the production of lupeol in strain LU-23 reached 280.46 mg/L in shake flasks. Further improvement was achieved through optimization of the carbon source in the medium (Additional file 3), and the final lupeol production reached 411.72 mg/L (Additional file 1: Fig. S3), which was 33.20-fold higher than that in the initial strain LU-6 and 9.67-fold higher than that in the control strain LU-9.

In summary, boosting the flux of the MVA pathway by overexpressing rate-limiting enzymes (*tHMG1, ERG1* and *ERG9*) only slightly enhanced lupeol biosynthesis. Lipid manipulation, however, had a marked effect on lupeol overproduction, especially the overexpression of *OLE1* and the disruption of *PAH1*-*DGK1*. These three modified strains and the control strain LU-9 (marked with asterisks in Fig. 2b) were selected for further investigations to reveal the mechanism underlying the high production.

### Effect of overexpression of *OLE1* and disruption of *PAH1*-*DGK1* on the UFA proportion and cell morphology

**Fig. 3:**
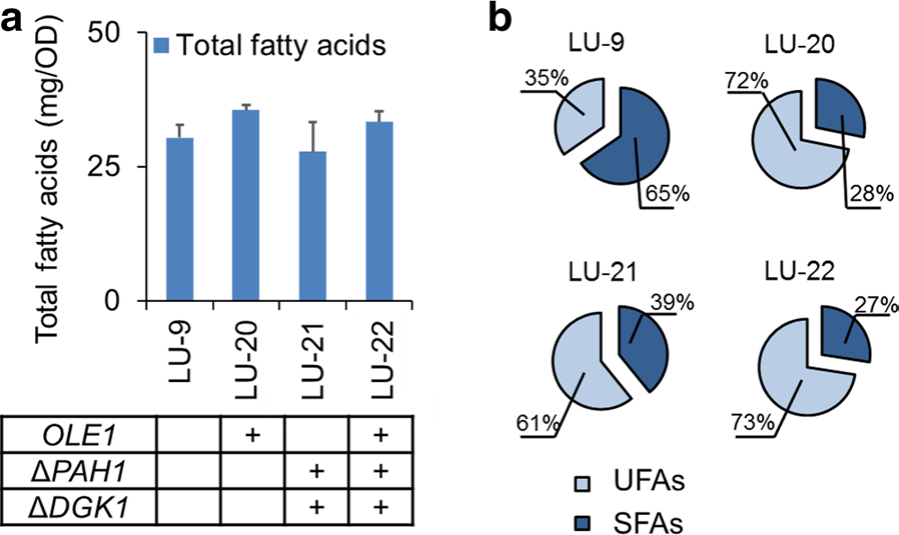
Effects of lipid manipulation on lipid content and composition. **a** Total fatty acid content. **b** Percentage of unsaturated fatty acids and saturated fatty acids. Error bars represent ± SD of technical triplicates

To elucidate the underlying relationship between lupeol production and lipid regulation, we measured the lipid content and the composition of UFAs and SFAs in high-production strains (LU-20, LU-21, and LU-22, marked with asterisks in Fig. 2b) and the control strain (LU-9). As shown in Fig. 3a, there was no obvious change in the total amount of fatty acids among the different strains. However, the UFA/SFA ratio was dramatically increased in all of the modified strains (Fig. 3b). The UFA content in the control strain (LU-9) was only 35%, but the UFA proportions in all the high-production strains increased up to 61–73%, approximately twofold higher than that in the control strain. These findings suggested that overexpressing *OLE1* and knocking out *PAH1*-*DGK1* mainly enhanced the proportion of UFAs instead of total fatty acid accumulation. The increased UFA proportion caused by *OLE1* overexpression and *PAH1*-*DGK1* knockout was consistent with previous studies [43–45]. The *OLE1* gene encodes a Δ9-fatty acid desaturase, which is mainly responsible for catalyzing the dehydrogenation of the 9-position in SFAs to form the related UFAs [43]. Moreover, knockout of *PAH1* could also result in increased levels of UFAs in yeast, even though this gene is responsible for phosphatidic acid (PA) transformation [44] (Additional file 1: Fig. S2).

**Fig. 4:**
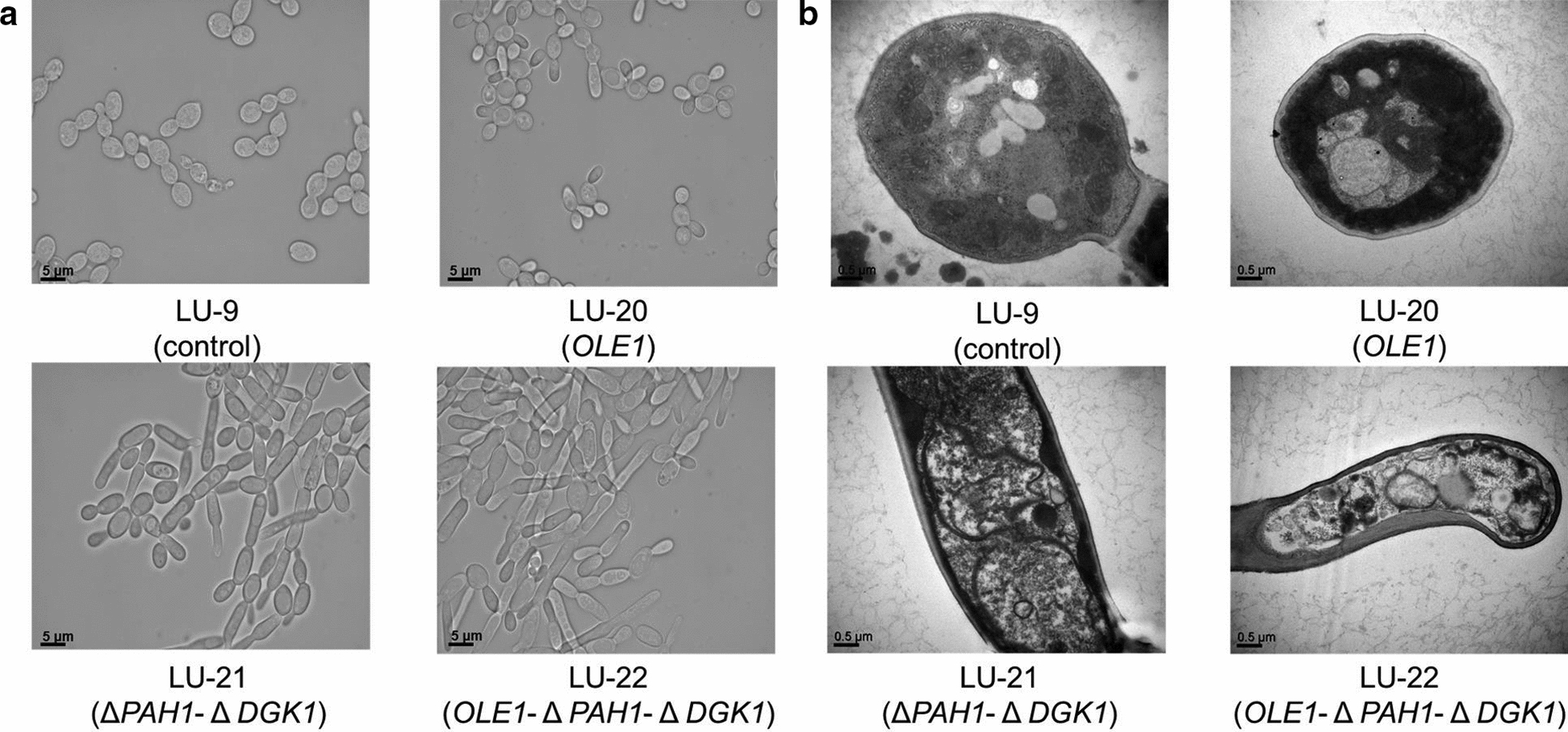
Cell morphology of strains LU-9, LU-20, LU-21 and LU-22. **a** Morphological analysis by optical microscopy (scale bars, 5 µm). **b** Morphological analysis by transmission electron microscopy (scale bars, 0.5 µm). The strain LU-20 with *OLE1* overexpression maintained a spherical morphology, which is similar to that of the control strain LU-9. However, disruption of *PAH1*-*DGK1* in LU-21 and LU-22 led to a distinctive elongated morphology

In addition to the change in lipid components, we also observed that the morphologic profiles of *PAH1*-*DGK1* knockout strains changed significantly. As detected by optical microscopy (Fig. 4a) and transmission electron microscopy (Fig. 4b), *PAH1*-*DGK1* knockout in strains LU-21 and LU-22 resulted in extraordinarily elongated morphology, while strain LU-20 with *OLE1* overexpression exhibited the same spherical cells as the control strain LU-9. The *PAH1* knockout was also able to generate massive proliferation of ER membranes, providing more binding sites for ER-anchored enzymes to promote terpene biosynthesis [13]. However, no obvious ER proliferation in strains LU-20 and LU-21 with *PAH1* knockout was observed by morphological analysis (Fig. 4b). As such, the effect of lipid modification on ER proliferation might not be the main factor associated with lupeol overproduction in this study.

**Fig. 5:**
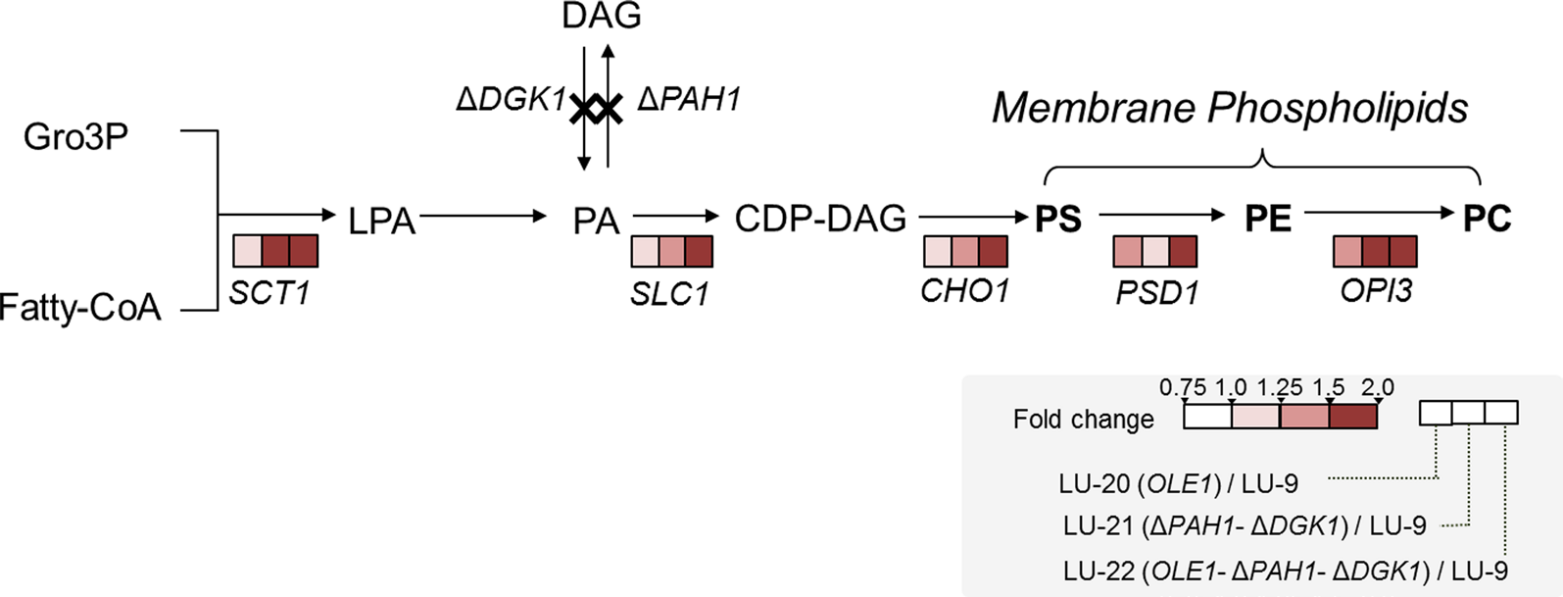
Transcriptomic analysis of genes related to membrane phospholipid synthesis. The color in each rectangle and circle represents the change ratio of FPKM. Gro3P, glycerol-3-phosphate; LPA, lysophosphatidic acid; CDP-DAG, cytosine diphosphate-diacylglycerol; PS, phosphatidyl serine; PE, phosphatidyl ethanolamine; PC, phosphatidyl choline

The *PAH1* gene encodes a phosphatidate phosphatase [44]. Single knockout of *PAH1* caused shortening of the chronological life span and severe cell lethality, which could be rescued by knocking out *DGK1* with *PAH1* synchronously [44–46]. The transformation from PA to DAG was blocked with *PAH1*-*DGK1* knockout, which could result in elevated levels of PA [44] and consequently lead to increased biosynthesis of phosphatidylserine (PS), phosphatidylethanolamine (PE), and phosphatidylcholine (PC) through the CDP-diacylglycerol (CDP-DAG) pathway [45]. We performed an RNA-seq analysis for the four strains, and as shown in Fig. 5, the genes related to the CDP-DAG pathway were obviously upregulated more than 1.50-fold in the *PAH1*-*DGK1*-deleted strain (LU-21 and LU-22) relative to the control strain (LU-9), including the genes encoding YALI0_C00209g (*SCT1*), YALI0_E18964g (*SLC1*), YALI0_D08514g (*CHO1*) and YALI0_E12441g (*OPI3*) (Additional file 2: Table S3). However, in the LU-20 strain, no similar upregulation of these genes was observed. The increased synthetic flux of PS, PE and PC, serving as the main components of membrane phospholipids, might result in accelerated synthesis of membrane phospholipids and is speculated to be the main factor underlying the elongated cell morphology of *PAH1*-*DGK1* disruption strains.

Previous studies have demonstrated that elongated cell morphology might also be an indicator that cells might be experiencing a stress response, such as toxic product accumulation or lipid oxidation with increased UFA content [47, 48]. To verify whether the cell morphology transition was induced by lupeol accumulation, we further analyzed the morphologic profiles of the strain by knocking out *PAH1*-*DGK1* in ATCC 201249, generating strain LU-29. As shown in Additional file 1: Fig. S4, the cell membrane of strain LU-29 was still elongated without lupeol accumulation. In addition, strain LU-20, with high UFA content (up to 72%), still exhibited spherical cells similar to those of the control strain LU-9. These results indicated that the elongated cell morphology was not induced by lupeol accumulation or UFA oxidation but rather by the genetic modification of lipid metabolism in this work.

In summary, *OLE1* overexpression and *PAH1*-*DGK1* disruption led to a marked change in lipid components. All three modified strains exhibited obviously increased proportions of UFAs instead of lipid accumulation. Knockout of *PAH1*-*DGK1* elongated the cell morphology, which might have been caused by the enhanced flux of membrane phospholipid synthesis.

### Effect of lipid modifications on the efflux of metabolites

**Fig. 6:**
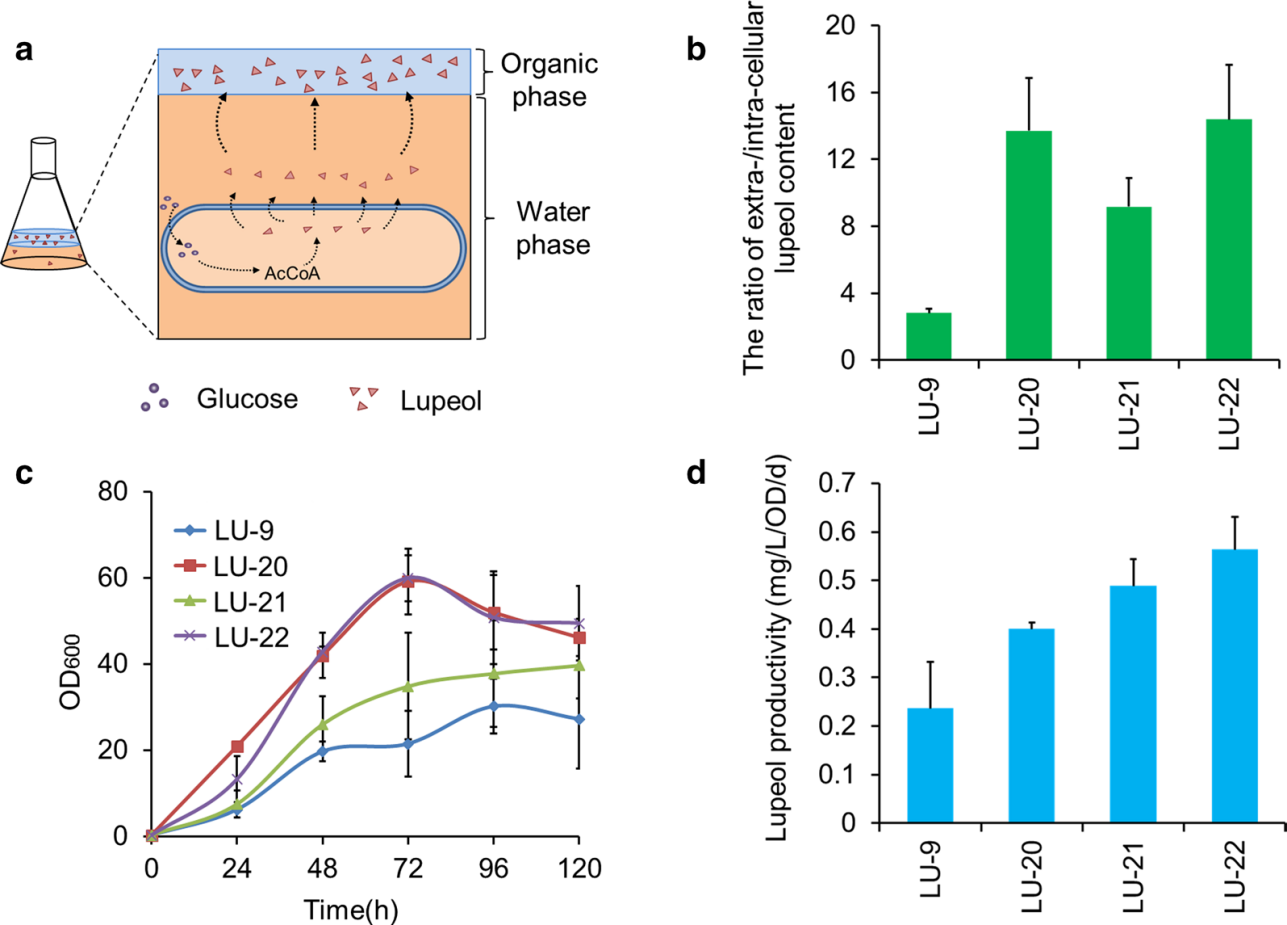
Effect of lipid modifications on the efflux of lupeol during two-phase fermentation. **a** Schematic representation of two-phase extractive fermentation. **b** Ratio of the extra/intracellular lupeol content after 120 h of fermentation, calculated based on the lupeol titer as shown in Additional file 1: Fig. S5. **c** Effect of lipid modifications on cell growth. **d** Lupeol productivity after 120 h of fermentation. These results indicated that the efflux of lupeol was enhanced and ultimately improved cell growth and the capacity of lupeol synthesis in engineered strains. Error bars represent ± SD of biological triplicates

Various studies have demonstrated that the increased UFA/SFA ratio disrupts the order of the phospholipid bilayer, contributing to the enhanced membrane permeability of toxic substances [19–24]. Meanwhile, the resulting cell elongation increased the contact area between the cells and the external environment. Both of the results described above are thought to have accelerated the efflux of products. To validate this hypothesis, we first tested the extra- and intracellular lupeol titers and analyzed the ratio of the extra/intracellular lupeol content in two-phase extraction fermentations of LU-9, LU-20, LU-21 and LU-22 (Fig. 6a, b and Additional file 1: Fig. S5). Compared to LU-9, the total lupeol production and the extracellular lupeol titer in the engineered strains increased significantly, while there was no obvious change in lupeol accumulation intracellularly (Additional file 1: Fig. S5). Similarly, as shown in Fig. 6b, the ratio of the extra/intracellular lupeol content also significantly increased to 13.71 in LU-20 with *OLE1* overexpression and 9.71 in LU-21 with *PAH1*-*DGK1* disruption, exhibiting 4.81- and 3.27-fold higher values than that in LU-9, in which the ratio was 2.81. An optimal ratio of 14.38 was achieved in LU-22 with combinatorial engineering, 5.11-fold higher than that in LU-9. These results indicated that the lipid modifications indeed resulted in improvement of product efflux. Then, we determined the biomass growth and cell integrity. As shown in Fig. 6c, engineered cells grew much faster in the logarithmic phase, and the biomass accumulation of strains LU-20, LU-21 and LU-22 was 2.76-, 1.62- and 2.79-fold higher than that of strain LU-9, respectively. Cell integrity was also improved, as the propidium iodide (PI) uptake factor decreased in the engineered strains (Additional file 1: Fig. S6 and Additional file 3). These results confirmed that the improvement of lupeol efflux was beneficial to cell growth and integrity, indicating that cytotoxicity was indeed reduced in the engineered strains. We then calculated the lupeol productivity after 120 h of fermentation. As shown in Fig. 6d and Additional file 2: Table S4, notable improvements in lupeol-specific productivity in the engineered strains were also observed. Compared to 0.22 mg/L/OD/days in strain LU-9, lupeol productivity increased to 0.40 mg/L/OD/days, 0.49 mg/L/OD/days and 0.56 mg/L/OD/days in LU-20, LU-21 and LU-22, respectively, indicating that the capacity of lupeol synthesis was also apparently increased in the engineered strains. Notably, the improved lupeol productivity was induced by single-cell lupeol synthesis capacity but not the improvement of biomass growth.

Collectively, these results confirmed that the elevated UFA/SFA ratio and elongated cells significantly enhanced the export of lupeol, which contributed to higher cell growth and integrity and consequently enabled higher lupeol synthesis capacity.

### Effect of lipid modifications on the production of other terpenoid classes

**Fig. 7:**
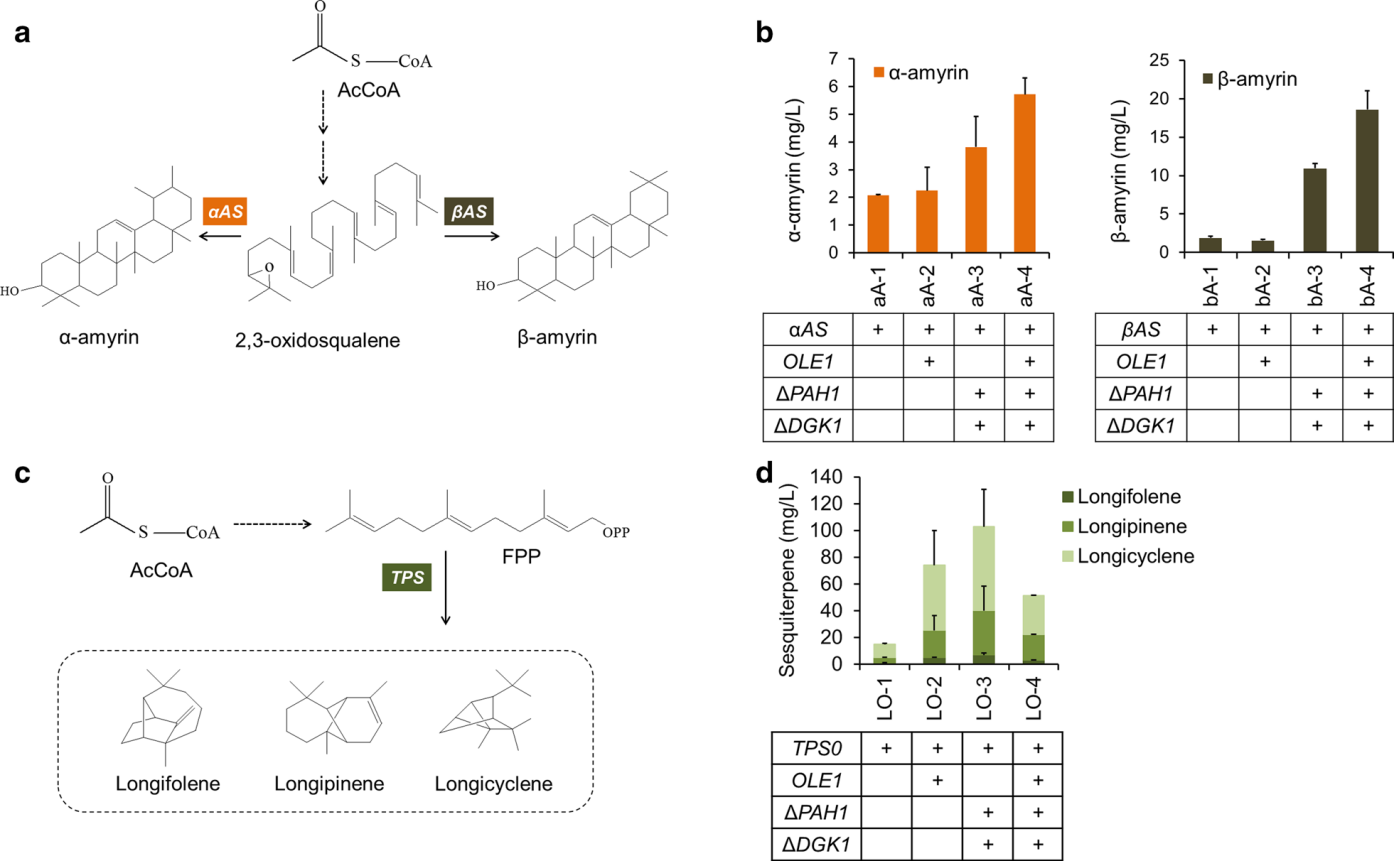
Production of other triterpenes and sesquiterpenes in the lipid-modified strain with *OLE1* overexpression and *PAH1*-*DGK1* knockout. **a** Schematic representation of the α-amyrin and β-amyrin biosynthesis pathways in *Y. lipolytica*. **b** Effect of lipid manipulation on the production of α-amyrin and β-amyrin. **c** Schematic representation of the biosynthetic pathway of the longifolene class of sesquiterpenes in *Y. lipolytica*, including longifolene, longipinene and longicyclene. **d** Effect of lipid manipulation on the production of the longifolene class of sesquiterpenoids. Error bars represent ± SD of biological triplicates

To test whether our strategy can be widely applied to enhance the production of other terpenoids, we first established the synthesis of two other kinds of triterpenoids (α- and β-amyrin) by expressing α-amyrin synthetase from *Malus domestica* (*αAS*) and β-amyrin synthetase from *A. thaliana* (*βAS*), respectively [49] (Fig. 7a). Positive effects of lipid metabolism modification on α-amyrin and β-amyrin production were obtained, with 1.76- and 8.83-fold improvement, respectively, in the titers compared to the parent strain (Fig. 7b). In addition to the triterpenoids, we further investigated the capacity of the lipid modifications for the production of sesquiterpenes. Longifolene class sesquiterpenoids, which serve as high-value therapeutic molecules, were employed [50] (Fig. 7c). The biosynthesis of longifolene, longipinene and longicyclene was realized by expressing terpene synthases (*TPS*) from *Pinus sylvestris* [51]. After 5 days of cultivation, obviously increased production was obtained in the lipid modification strain LO-3, with 5.68-fold higher sesquiterpene production compared to the parent strain LO-1 (Fig. 7d).

## Discussion

As a pentacyclic triterpenoid, lupeol has valuable pharmaceutical and nutraceutical properties [3, 8, 30], but a severely negative impact of lupeol on cell viability was observed even at a relatively low concentration of 60 mg/L [3]. *Y. lipolytica* is emerging as a fascinating host for terpenoid biosynthesis, but the cytotoxicity of terpenes induced by their antimicrobial activities is still a main factor hampering terpene overproduction in *Y. lipolytica* [4, 5].

To improve lupeol production, a classic strategy that mainly boosts the flux of the MVA pathway was first employed by overexpressing rate-limiting enzymes (*tHMG1, ERG1 and ERG9*). However, lupeol production exhibited slight improvement, which is consistent with the results of the production of amorphadiene in *Y. lipolytica* [35]. In addition to the MVA pathway modifications, lipid manipulation was proven to be another promising alternative to accelerate the storage capacity for lipophilic products and ultimately promote their overproduction [9–14]. Increasing the UFA content in lipid droplets contributed to improving the solubility of highly lipophilic tetraterpenoids (such as lycopene and β-carotene) in oil droplets and thereby accelerated the intracellular accumulation of large-molecule terpenes [11, 14]. In contrast to previous studies, we creatively accelerated the efflux of relatively small molecular terpenes by altering lipid metabolism via *OLE1* overexpression and *PAH1*-*DGK1* knockout. The resulting increase in the UFA/SFA ratio contributed to enhancing the membrane permeability of toxic substances [52] and potentially increasing the extra/intracellular partition coefficient of terpenoids with a highly nonpolar nature in the IPM phase [15].

Previous studies have demonstrated that the oxidation of UFAs or toxic chemical accumulation might induce a stress response and facilitate the cell morphology transition into a filamentous stage, consequently reducing cell viability and cell growth [47, 48]. However, the reason for the elongated cell morphology in this study was not lupeol accumulation, as the elongated cell morphology was still present in strain LU-29, which is a *PAH1*-*DGK1* knockout strain that lacks the lupeol synthesis pathway. Cell elongation was not caused by oxidative stress with increasing UFA content because strain LU-20, with a high UFA content (72%), exhibited the same spherical cells as the control strain LU-9. Moreover, the developed cell growth and improved cell integrity of the engineered strains also indicated that the cells did not experience a stress response. Therefore, the elongated cell morphology was the result of lipid metabolism via *PAH1*-*DGK1* knockout.

## Conclusions

In this study, we developed a strategy to create a high-yield lupeol-producing strain via regulation of lipid components. Lipid manipulation significantly accelerated terpene excretion, enhancing the terpene synthesis capacity. The final lupeol production reached 411.72 mg/L in shake flasks, which is the highest lupeol yield reported to date. In addition, our strategy was also valid for overproduction of other triterpenes and sesquiterpenes. This work provides a novel and general strategy to improve the biosynthesis of valuable but toxic terpene products in microbial cell factories.

## Methods

### Strains, media and culture conditions

The *Y. lipolytica* strain ATCC 201249 (*MATA, ura3*-*302, leu2*-*270, lys8*-*11, PEX17*-*HA*) was chosen as the background strain [53] for all constructs, and the genotypes of all the derivatives constructed in the present study are listed in Additional file 2: Table S5. The above *Y. lipolytica* strains were cultivated in yeast medium at 28 °C at a shaking speed of 250 rpm. The rich YPD medium containing 50 g/L glucose, 20 g/L peptone, and 10 g/L yeast extract was used for cultivation and fermentation of *Y. lipolytica* strains. The SC medium, used for screening *Y. lipolytica* transformants, contained 20 g/L glucose, 6.7 g/L yeast nitrogen base without amino acids, and 2 g/L complete supplement mixture (CSM) lacking uracil (SC-Ura) or leucine (SC-Leu), supplemented with uracil or leucine depending on the selection marker requirements. If necessary, an appropriate amount of hygromycin B (final concentration 100 mg/L) was added at 50–60 °C for screening of recombinant strains containing the hygromycin resistance gene (Hph). *DH5α E. coli* was used for routine plasmid propagation and construction of recombinant vectors and grown in Luria–Bertani broth at 37 °C with a shaking speed of 250 rpm. Suitable antibiotics were added when necessary at the following final concentrations: ampicillin at 100 mg/L or kanamycin at 50 mg/L. Agar (20 g/L) was added for solid plate preparation.

For terpene shake flask fermentation, freshly streaked single colonies of strains were first cultivated in 25-mL polypropylene tubes with 5 mL of YPD medium and cultured at 28 °C with a shaking speed of 250 rpm overnight. After preculturing, the seed cultures were inoculated into 50 mL of fresh YPD medium with an initial OD_600_ of 0.2 and fermented in a 250-mL shake flask for 120 h. Ten milliliters of isopropyl myristate (IPM) was added for two-phase extraction fermentation to reduce the toxicity inhibition of steroid products on cell growth. All flask fermentations were performed in triplicate.

### Strain and plasmid construction and yeast transformation

All plasmids and primers applied in this work are listed in Additional file 2: Tables S6 and S7, respectively. All the enzymes involved in this study were obtained from New England Biolabs (NEB, USA). The TIANprep Mini Plasmid Kit and TIANgal Midi Purification Kit were used for plasmid extraction and DNA fragment purification, respectively, and the Zymogen Frozen EZ Yeast Transformation Kit II (Zymo Research Corporation) was employed for *Y. lipolytica* transformation. The relevant processes were carried out in accordance with the manufacturer’s instructions.

Six exogenous lupeol synthase-coding genes (*AtLus* from *A. thaliana*, *GuLus* from *G. uralensis*, *OeLus* from *O. europaea*, *BgLus* from *B. gymnorhiza*, *KdLus* from *K. daigremontiana* and *RcLus* from *R. communis*) were codon-optimized and synthesized by GenScript (Nanjing, China) (codon-optimized sequences in Additional file 2: Table S1). Three native genes in the MVA pathway, namely *HMG1* (3-hydroxy-3-methylglutaryl coenzyme A reductase), *ERG9* (squalene synthase), and *ERG1* (squalene monooxygenase), and three native genes in the lipid metabolism pathway, namely, *ACC1* (acetyl-CoA carboxylase from *Y. lipolytica*), *OLE1* (Δ9-fatty acid desaturase) and its activator *MGA2* substitution *MGA2*^*G643R*^, were amplified from *Y. lipolytica* genomic DNA by a normal polymerase chain reaction (PCR) method using the primers listed in Additional file 2: Table S7. All of these genes were cloned into the expression plasmids at the corresponding sites with different promoters and the LEU2, URA3 or Hph marker. The resulting recombinant plasmids were digested with *NotI* and purified on a gel. Then, approximately 2 μg of linearized DNA was used in the transformation reaction, and the transformants were centrifuged at 6000×*g* for 2 min, plated on SC agar plates without the auxotrophic compound supplemented by the corresponding markers, and cultured at 28 °C for 2–3 days. Positive transformants were confirmed by colony PCR with KOD FX DNA polymerase (Toyobo Co., Ltd.; Shanghai, China). Removal of the Ura3 selection marker was carried out by shaking the transformants in YPD liquid medium for 2–3 days and then incubating the transformants on YPD solid medium containing 1.2 mg/mL 5-fluoroorotic acid for 2 days. The obtained colonies were then streaked onto SC and SC-Ura plates and incubated at 28 °C for 2–3 days.

Disruption of target genes in *Y. lipolytica* was achieved by the CRISPR system [27], which has been widely used in various studies. The plasmid pMCS-Cen1 was used to construct the CRISPR–Cas9 system. The synthesized gRNA was incorporated into pMCS-Cen1-URA, and then, the resulting plasmids were digested with the restriction enzymes *BamHI* and *HindIII* followed by ligation with a segment of Cas9, thereby forming the corresponding plasmids. For CRISPR plasmid transformations, *Y. lipolytica* cells were transformed with the corresponding plasmids and cultivated in SC-Ura liquid medium for 4 days. Then, the cells were plated onto SC-Ura plates and cultured for 2 days at 28 °C and confirmed via sequencing analysis.

### Extraction and analysis of triterpenoids (lupeol, α-amyrin, and β-amyrin) and sesquiterpenes (longifolene, longicyclene and longipinene)

The extraction of triterpenes (lupeol, α-amyrin, β-amyrin) and sesquiterpene products (longifolene, longifolene, longifolene) is mainly divided into organic-phase and cell-phase extraction. For the organic-phase sample, the two-phase fermentation broth was centrifuged at 5000 rpm for 5 min, and then, the supernatant was filtered by a 0.22-µm polypropylene organic filter for extracellular terpenoid detection. The precipitate was prepared for the extraction of cell-phase samples. After centrifugation and washing 3 times with distilled water to ensure adequate removal of the residual organic phase, the precipitate was mixed with 0.5 mL of ethyl acetate and 0.1 mL of quartz sand and then vortexed for 20 min. After centrifugation at 15,000 rpm for 15 min, the organic mixture was filtered through a 0.22-µm organic filter membrane. Appropriate concentrations of lupeol (Aladdin Industrial Corporation, USA), α-amyrin, β-amyrin (Sigma-Aldrich Corporation, USA) and longicyclene (Shanghai Yuanye Bio-technology Corporation, China) were dissolved in IPM as external standards to ensure the production of terpenoids. All of the final samples in two phases were stored at − 80 °C.

The methods for the analysis of terpenoids by gas chromatography–mass spectrometry (GC–MS) were modified based on those reported in a previous study [37]. The combination of a GC–MS system equipped with a DB-5MS GC column (30 m × 0.25 mm × 0.5 μm) and Masslynx software (Version 4.1, Waters Corp., USA) was applied for qualitative and quantitative analysis of triterpenoids. The details of the analysis process are as follows. For the GC system, 1 µL of the obtained sample was injected by an Agilent 7683 autosampler under a split ratio of 10, and the temperature of the injector and GC interface was 250 °C. The carrier gas was high-purity helium with a constant flow of 1.2 mL/min. After 2 min at 80 °C, the column temperature was increased at a speed of 20 °C/min to 300 °C, holding at this temperature for 17 min. For the MS system, the temperature of ion source was 230 °C, and full scan mode was used from 50 to 700 *m/z*.

For the analysis of sesquiterpenes, some parameters were adjusted as follows. For the GC system, the split ratio and the temperature of the injector and GC interface were adjusted to 50 and 250 °C, respectively. The carrier gas was high-purity helium with a constant flow of 2 mL/min. After 2 min at 40 °C, the column temperature was increased to 210 °C at a speed of 20 °C/min, followed by increasing to 300 °C at a speed of 60 °C/min and holding for 2 min. For the MS system, the ion source temperature was set at 220 °C, and the scanning range parameter was 35–500 *m/z*.

### Extraction and analysis of total fatty acids

For total fatty acid extraction, fresh samples of cell cultures were harvested after 120 h of fermentation (three duplicates). After centrifugation and washing 3 times with distilled water, 1 mL of methanol solution containing 3 M hydrogen chloride, 0.1 mL of chloroform and 5 μL of heptadecanoic acids as an internal standard (final concentration of 200 mg/L) was added into the precipitate, followed by incubation at 70 °C for 3 h, mixing by inversion every 40 min. After naturally cooling the samples to room temperature, 0.2 mL of sodium chloride particles was added, and the samples were vortexed for 1 min, followed by the addition of 0.5 mL of the organic solvent *n*-hexane and vortexing for 3 min. After centrifugation at 12,000 rpm for 5 min, the upper organic phase was filtered with a 0.22-μm organic film and stored at − 80 °C, ready for the detection of total fatty acid concentration by GC–MS.

The method used for the analysis of total fatty acids by GC–MS was similar to the methods reported previously [54]. For the GC system, 1 µL of the obtained sample was injected by an Agilent 7683 autosampler under a split ratio of 2, and the temperatures of the injector and GC interface were both 280 °C. The carrier gas was high-purity helium with a constant pressure of 91 kPa. After 2 min at 70 °C, the column temperature was increased at a speed of 8 °C/min to 290 °C, holding at this temperature for 6 min. For the MS system, the ion source temperature was set at 250 °C, and the scanning range parameter was 50–800 *m/z*.

### RNA-seq analysis

Culture samples were collected at logarithmic phase (two parallel measurements) after centrifugation at 5000 rpm for 2 min, washed with PBS solution 3 times and quickly frozen with liquid nitrogen after centrifugation. The acquisition, analysis and sequencing of mRNA from the above samples were performed by Beijing Genomics Institute (BGI). Library construction was performed using cDNA, which was generated from mRNA after fragmentation and reverse transcription by N6 primers. Then, the library quality was determined using a Bioanalyzer 2100 (Agilent) analyzer, and the libraries were sequenced on the BGISEQ-500 sequencing platform. The sequencing reads were filtered and stored in FASTQ format. The relative expression of genes was calculated using RSEM software and the FPKM method. Finally, in-depth data analysis was performed on the BGI data analysis platform (http://report.bgi.com/ps/login/login.html).

### Morphological analysis by transmission electron microscopy

Cells were harvested, and the supernatant was discarded after centrifugation at 5000 rpm for 2 min, followed by resuspension and fixation with 2.5% glutaraldehyde solution overnight (4 °C). The mixture was rinsed three times with 0.1 M PBS for 15 min, fixed with 1% osmic acid for 1 to 2 h and then rinsed three times with 0.1 M PBS. Then, the obtained sample was dehydrated by ethanol solutions with concentrations of 30%, 50%, 70%, 80%, 90%, and 95%, successively, and then treated as follows: 100% ethanol for 20 min, acetone solution for 20 min, acetone-embedding agent (*V*/*V* = 1/1) mixed solution for 1 h, acetone-embedding agent (*V*/*V* = 3/1) mixed solution for 3 h, and pure embedding agent for 12 h. After heating at 70 °C for 12 h, the embedded sample was sectioned with a Leica EM UC7 ultrathin microtome (70–90 nm), stained with lead citrate and 50% uranyl acetate ethanol solution for 5 min, and then observed with a transmission electron microscope.

## Supplementary information

**Additional file 1: Fig. S1.** Optimization of the heterologous lupeol synthesis pathway in *Y. lipolytica*. (A) Relative lupeol production by lupeol synthases from different sources. (B) Lupeol production by the identified gene *RcLus* under the control of different promoters. (C) Microscopic images of strains modified with the *sfGFP* gene by fusing different subcellular localization signal peptides. Cells were observed by laser scanning confocal microscopy. (D) Effect of different subcellular locations on lupeol production. Abbreviations: Mit, mitochondria; Per, peroxisome; ER, endoplasmic reticulum. Error bars represent ± SD of technical triplicates. **Fig. S2.** Schematic representation of key genes associated with lipid metabolism in *Y. lipolytica*. All genes in blue rectangles were regulated in this study. Red arrows represent relative pathway upregulation, and red crosses represent relative pathway deletion. Red circles indicate mutual inhibition between genes. Green circles indicate mutual promotion between genes. **Fig. S3.** Culture optimization for LU-23 lupeol production in shake flasks. (A) Effect of carbon optimization and (B) heterologous addition of pyruvic acid on lupeol yield. Error bars represent ± SD of technical triplicates. **Fig. S4.** Cell morphology of strain LU-29. Morphological analysis by optical microscopy (scale bars, 5 µm). The strain LU-29 without the lupeol synthesis pathway also exhibited a distinctive elongated morphology, indicating that the cell morphology transition had little relation with lupeol accumulation. **Fig. S5.** Extracellular and intracellular lupeol titers after 120 h of fermentation. Compared with LU-9, the total lupeol production and the extracellular lupeol titer increased significantly in the engineered strains, while there was no obvious change in intracellular lupeol accumulation. These results indicated that the lupeol was discharged out of the cell more efficiently. Error bars represent ± SD of biotechnical triplicates. **Fig. S6.** Cell integrity in the stationary phase after 120 h of fermentation. Cell integrity was measured by the propidium iodide (PI) staining method, in which as cell integrity increases, PI uptake decreases. Compared to the control strain LU-9, all of the modified strains exhibited improved cell integrity with decreased PI uptake. Error bars represent ± SD of biotechnical triplicates.

**Additional file 2: Table S1.** Codon-optimized gene sequences involved in this study. **Table S2.** Subcellular targeting signal peptides used in this study. **Table S3.** Upregulated genes in the membrane phospholipid synthesis pathway. **Table S4.** Lupeol yield and productivity in selected strains. **Table S5.** Strains used in this study. **Table S6.** Plasmids used in this study. **Table S7.** Primer sequences used in this study.

**Additional file 3: Methods.** Culture optimization and cell integrity analysis.

## Data Availability

Data will be made available from the corresponding author on reasonable request.
