# Supplementary figures and images for: High production of triterpenoids in Yarrowia lipolytica through manipulation of lipid components
Source: Biotechnol Biofuels. 2020 Jul 29;13:133. doi: 10.1186/s13068-020-01773-1 (PMC7392732; doi:10.1186/s13068-020-01773-1)

**Additional file 1.**

**
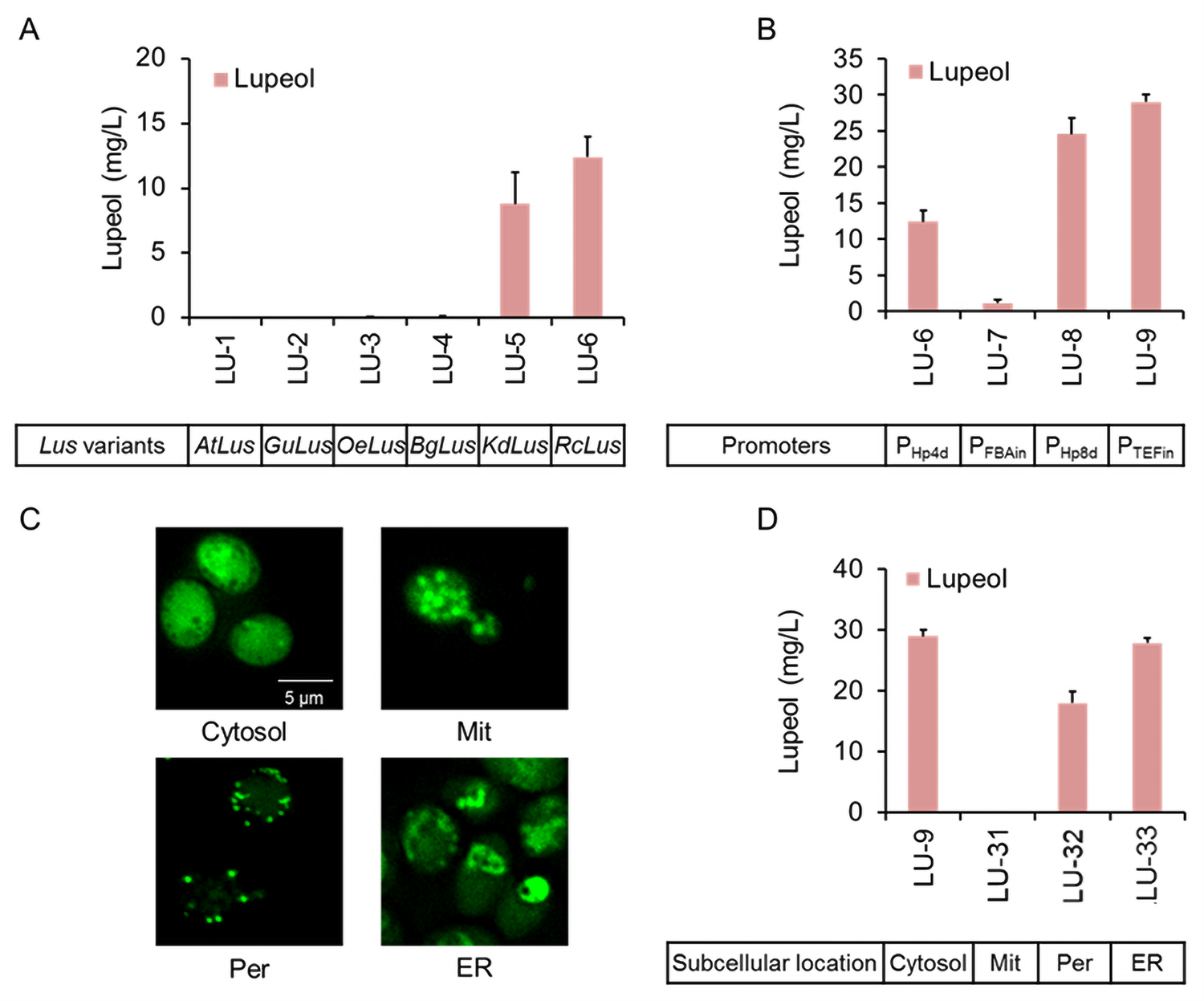
**

**Fig. S1**


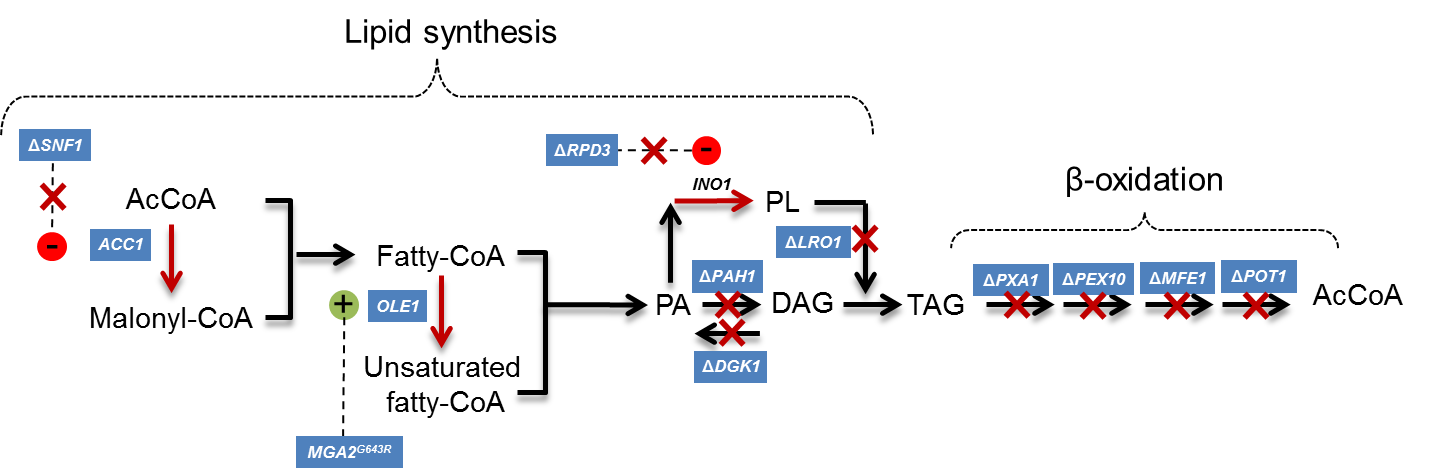


**Fig. S2**


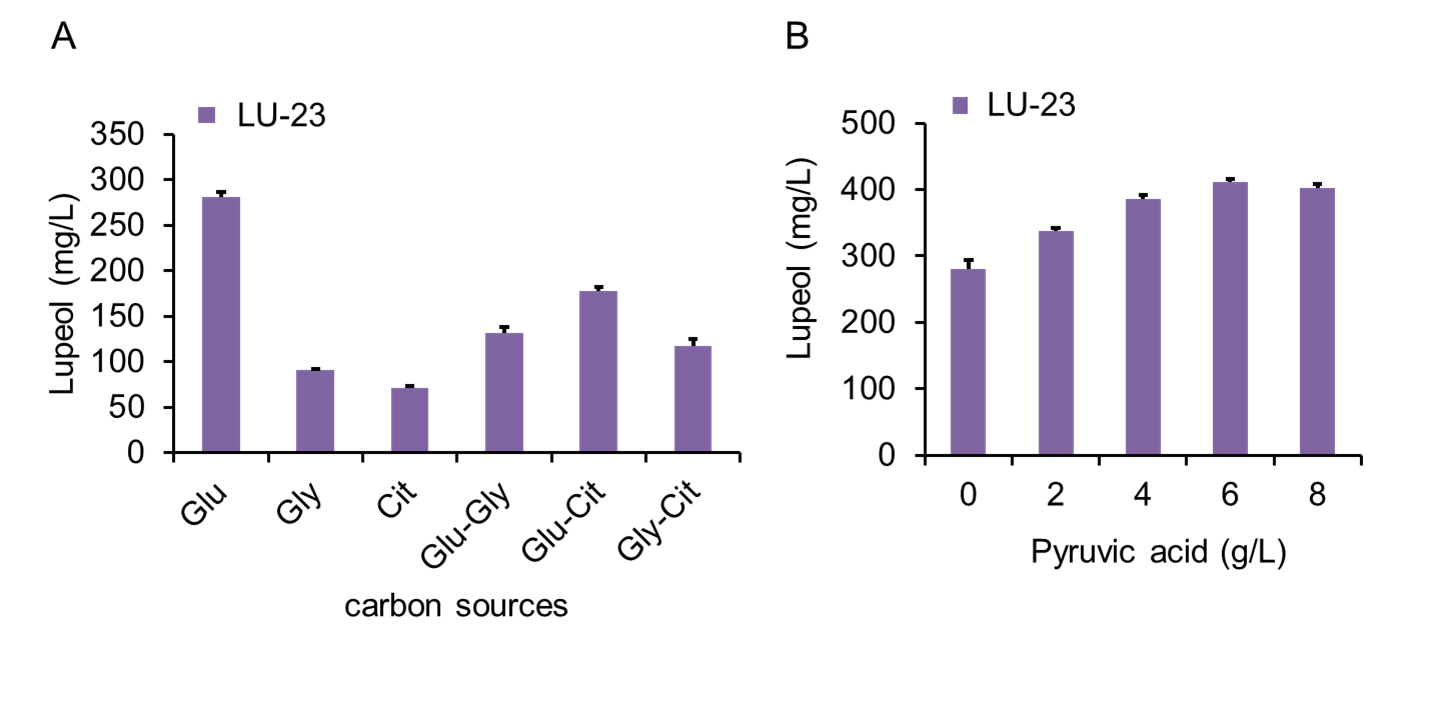


**Fig. S3**


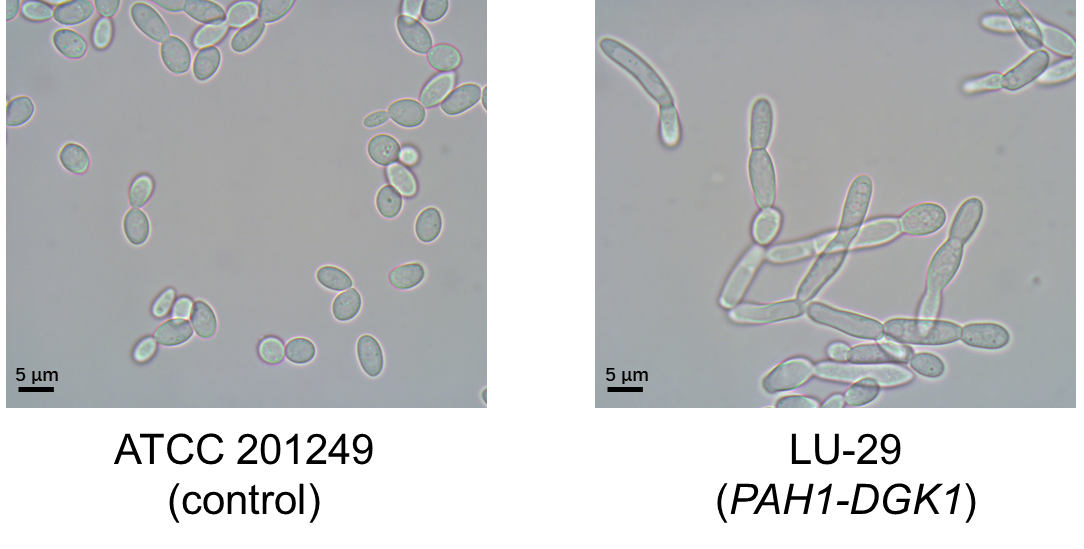


**Fig. S4**


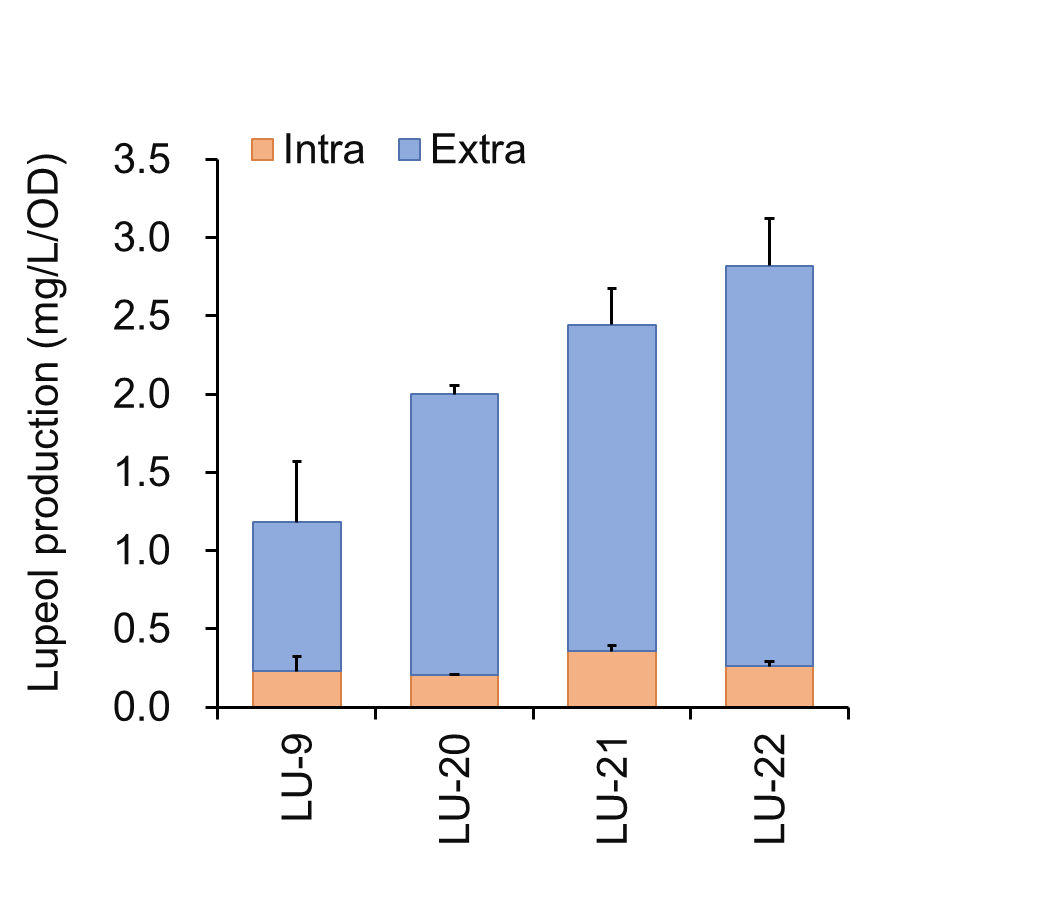


**Fig. S5**


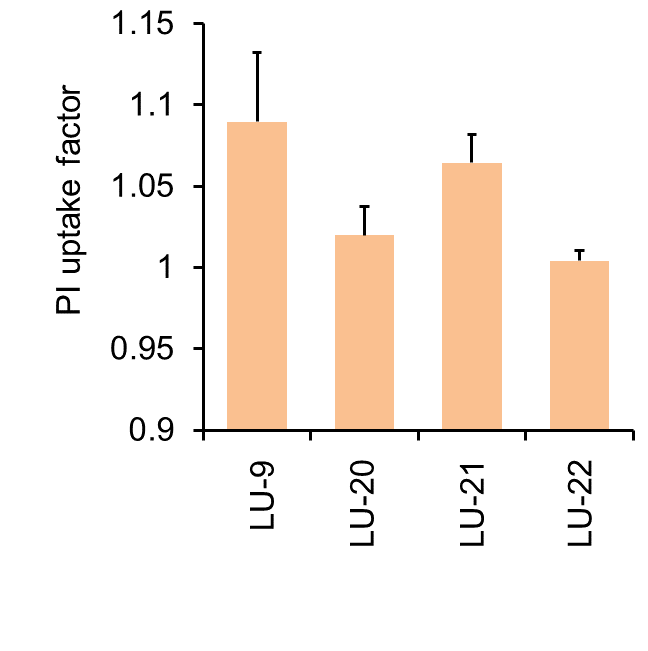


**Fig. S6**

Supplement: Supplementary file 1 — Additional file 1: Fig. S1. Optimization of the heterologous lupeol synthesis pathway in Y. lipolytica. (A) Relative lupeol production by lupeol synthases from different sources. (B) Lupeol production by the identified gene RcLus under the control of different promoters. (C) Microscopic images of strains modified with the sfGFP gene by fusing different subcellular localization signal peptides. Cells were observed by laser scanning confocal microscopy. (D) Effect of different subcellular locations on lupeol production. Abbreviations: Mit, mitochondria; Per, peroxisome; ER, endoplasmic reticulum. Error bars represent ± SD of technical triplicates. Fig. S2. Schematic representation of key genes associated with lipid metabolism in Y. lipolytica. All genes in blue rectangles were regulated in this study. Red arrows represent relative pathway upregulation, and red crosses represent relative pathway deletion. Red circles indicate mutual inhibition between genes. Green circles indicate mutual promotion between genes. Fig. S3. Culture optimization for LU-23 lupeol production in shake flasks. (A) Effect of carbon optimization and (B) heterologous addition of pyruvic acid on lupeol yield. Error bars represent ± SD of technical triplicates. Fig. S4. Cell morphology of strain LU-29. Morphological analysis by optical microscopy (scale bars, 5 µm). The strain LU-29 without the lupeol synthesis pathway also exhibited a distinctive elongated morphology, indicating that the cell morphology transition had little relation with lupeol accumulation. Fig. S5. Extracellular and intracellular lupeol titers after 120 h of fermentation. Compared with LU-9, the total lupeol production and the extracellular lupeol titer increased significantly in the engineered strains, while there was no obvious change in intracellular lupeol accumulation. These results indicated that the lupeol was discharged out of the cell more efficiently. Error bars represent ± SD of biotechnical triplicates. Fig. S6. Cel [file 13068_2020_1773_MOESM1_ESM.doc]
