# Supplementary material for: High production of triterpenoids in Yarrowia lipolytica through manipulation of lipid components
Source: Biotechnol Biofuels. 2020 Jul 29;13:133. doi: 10.1186/s13068-020-01773-1 (PMC7392732; doi:10.1186/s13068-020-01773-1)
Supplement: Supplementary file 2 — Additional file 2: Table S1. Codon-optimized gene sequences involved in this study. Table S2. Subcellular targeting signal peptides used in this study. Table S3. Upregulated genes in the membrane phospholipid synthesis pathway. Table S4. Lupeol yield and productivity in selected strains. Table S5. Strains used in this study. Table S6. Plasmids used in this study. Table S7. Primer sequences used in this study. [file 13068_2020_1773_MOESM2_ESM.doc]

**Additional file 2.**

**Table S1. Codon‑optimized gene sequences involved in this study.**

> *AtLus* from *Arabidopsis thaliana*

ATGTGGAAGCTGAAGATCGGCAAGGGCAACGGAGAAGACCCTCACCTGTTCTCTTCTAACAACTTCGTGGGCCGACAGACCTGGAAGTTCGACCACAAAGCCGGCTCTCCCGAAGAAAGAGCCGCTGTGGAGGAAGCTCGAAGAGGCTTTCTGGACAACCGATTCCGAGTGAAGGGCTGCTCTGATCTGCTGTGGCGAATGCAGTTCCTGCGAGAGAAGAAGTTCGAGCAGGGCATCCCTCAACTGAAGGCCACCAACATCGAGGAGATCACCTACGAGACTACCACTAACGCTCTCAGACGAGGCGTGCGATATTTCACCGCTCTGCAGGCTTCTGATGGACATTGGCCCGGAGAAATCACTGGCCCCCTGTTTTTTCTGCCTCCCCTGATCTTCTGCCTGTACATCACCGGCCATCTGGAGGAAGTGTTCGATGCCGAGCACCGAAAAGAGATGCTGCGACACATCTACTGCCACCAGAACGAAGATGGCGGATGGGGACTGCACATCGAGTCTAAGTCTGTGATGTTCTGCACCGTGCTGAACTACATCTGCCTGCGAATGCTGGGCGAAAACCCCGAACAAGACGCCTGCAAGAGAGCCAGACAGTGGATTCTTGATAGAGGCGGCGTGATCTTTATTCCCTCTTGGGGCAAGTTCTGGCTGTCTATCCTGGGCGTGTACGATTGGTCTGGCACTAATCCTACCCCTCCCGAACTTCTGATGCTGCCCTCTTTCCTGCCTATCCACCCCGGAAAGATCCTGTGCTACTCTCGAATGGTGTCTATCCCCATGTCTTACCTGTACGGCAAGAGATTCGTGGGACCCATCACTCCTCTGATCCTGCTGCTTCGAGAGGAGCTGTACCTGGAACCCTACGAGGAGATCAACTGGAAGAAGTCTCGACGACTGTACGCCAAGGAGGACATGTACTACGCTCACCCCCTGGTGCAAGATCTGCTGTCTGACACCCTGCAGAACTTTGTGGAGCCCCTGCTTACTCGATGGCCCCTGAATAAGCTGGTGCGAGAGAAGGCTCTGCAGCTGACCATGAAGCACATCCACTACGAGGACGAGAACTCTCACTACATCACCATCGGCTGCGTGGAGAAGGTTCTGTGCATGCTGGCTTGTTGGGTGGAGAATCCCAACGGCGACTACTTCAAGAAGCACCTGGCCCGAATCCCTGACTACATGTGGGTTGCTGAGGACGGCATGAAGATGCAGTCTTTCGGCTGCCAGCTTTGGGATACCGGCTTTGCCATCCAAGCTCTGCTGGCCTCTAATCTGCCCGACGAGACTGATGACGCCCTGAAACGAGGCCACAACTACATCAAGGCCTCTCAGGTGCGAGAAAACCCCTCTGGCGACTTCCGATCTATGTACCGACACATCTCTAAGGGCGCCTGGACCTTTTCTGACCGAGATCACGGATGGCAGGTGTCTGATTGCACCGCTGAGGCTCTGAAGTGTTGCCTGCTGCTGTCTATGATGTCTGCCGACATCGTGGGCCAGAAGATCGATGACGAGCAGCTGTACGACTCTGTGAACCTGCTGCTGTCTCTGCAGTCTGGCAATGGCGGAGTGAACGCTTGGGAACCCTCTAGAGCCTACAAGTGGCTGGAGCTGCTGAATCCCACCGAGTTCATGGCCAACACCATGGTGGAGCGAGAGTTCGTGGAATGCACCTCTTCTGTGATCCAGGCCCTGGACCTGTTCAGAAAGCTGTACCCCGACCACCGAAAGAAGGAGATCAACCGATCTATCGAGAAGGCCGTGCAGTTCATCCAGGACAACCAGACTCCTGACGGCTCTTGGTATGGCAATTGGGGCGTGTGCTTCATTTACGCCACCTGGTTTGCTCTTGGAGGACTGGCTGCTGCTGGAGAGACTTATAACGACTGCCTGGCCATGAGAAATGGCGTGCATTTCCTGCTTACTACCCAGCGAGATGATGGAGGATGGGGAGAGTCTTACCTGTCTTGCTCTGAGCAGCGATACATCCCTTCTGAGGGCGAGAGATCTAACCTGGTGCAGACCTCTTGGGCTATGATGGCCCTGATTCACACTGGACAGGCCGAACGAGATCTGATCCCCCTTCACAGAGCTGCCAAGCTGATCATCAACTCTCAGCTGGAGAACGGAGATTTCCCCCAGCAGGAAATTGTGGGCGCCTTCATGAACACCTGCATGCTGCACTACGCCACCTACCGAAACACCTTTCCCCTGTGGGCTCTGGCCGAGTATCGAAAGGTGGTGTTCATCGTGAACTAA

*> GuLus* from *Glycyrrhiza uralensis*

ATGTGGAAGCTGAAGATTGGCGAAGGCGGAGCTGGACTGATCTCTGTGAACAACTTCATCGGCCGACAGCACTGGGAGTTCGATCCTAATGCCGGCACTCCTCAGGAACATGCCGAGATCGAACGACTGCGACGAGAGTTCACCAAGAACCGATTCTCTATCAAGCAGTCTGCCGACCTGCTGATGAGAATGCAGCTGCGAAAGGAGAACCACTACGGCACCAACAACAATATTCCCGCCGCCGTGAAATTATCTGACGCCGAGAACATCACTGTGGAGGCTCTGGTGACTACTATCCGACGAGCCATCTCTTTCTACTCTTCTATCCAGGCCCATGACGGACACTGGCCTGCTGAATCTGCTGGCCCCCTGTTTTTTCTGCAGCCCCTGGTGATGGCTCTTTACATCACCGGCTCTCTGGATGATGTTCTGGGCCCTGAGCACAAAAAGGAGATCGTGCGATACCTGTACAACCACCAGAATGAGGACGGAGGATGGGGCTTCCATATTGAGGGCCACTCTACCATGTTTGGCTCTGCCCTGTCTTATGTGGCCCTGAGAATCTTAGGCGAAGGCCCCGAAGATAAAGCTATGGCCAAGGGCCGAAAGTGGATTCTGGATCACGGCGGACTGGTGGCTATTCCTTCTTGGGGCAAGTTCTGGGTGACTGTGCTGGGCGCTTATGAATGGTCTGGCTGCAACCCTCTGCCTCCTGAACTGTGGCTGCTGCCTAAGTTCACCCCCTTCCACCCTGGAAAGATGCTGTGCTACTGCCGACTGGTGTACATGCCCATGTCTTACCTGTACGGCAAGAAGTTCGTGGGCCCTATCACCGCCCTGATTCGATCTCTGCGAGAGGAGCTGTACAACGAGCCCTACAACCAGATCAACTGGAACACCGCCCGAAACACCGTTGCTAAGGAGGACCTGTATTACCCCCACCCCCTGATTCAAGACATGCTGTGGGGCTTCCTGTATCATGTGGGCGAGCGATTTCTTAACTGCTGGCCCTTCTCTATGCTGCGACGAAAGGCTCTGGAGATCGCCATCAACCACGTCCACTACGAGGACGAGAACTCTCGATACCTGTGCATCGGCTCTGTGGAGAAGGTGCTGTGTCTGATCGCCAGATGGGTGGAGGACCCCAATTCTGAGGCCTACAAGCTGCACCTTGCCCGAATTCCCGACTACTTCTGGCTTGCTGAGGACGGACTGAAGATCCAGTCTTTCGGCTGCCAGATGTGGGATGCCGCCTTTGCTATCCAAGCTATCCTGGCCTGAACGTGTCTGAAGAGTACGGACCCACCCTGAGAAAGGCTCACCACTTCGTGAAAGCCTCTCAGGTGCGAGAAAACCCCTCTGGAGACTTCAACGCCATGTACCGACACATCTCTAAGGGCGCCTGGACCTTCTCTATGCACGACCACGGCTGGCAAGTGTCTGATTGCACAGCCGAGGGACTGAAAGCTGCTCTGCTGCTGTCTGAGATGCCCTCTGAACTGGTGGGCGGAAAGATGGAGACTGAGCGATTCTACGACGCCGTGAACGTGATCCTGTCTCTGCAGTCTTCTAACGGCGGCTTCCCTGCTTGGGAACCCCAAAAGGCCTATCGATGGCTGGAGAAGTTCAACCCCACCGAGTTCTTCGAGGACACCATGATCGAGCGAGAGTACGTGGAATGTACCGGCTCTGCCATGCAAGGACTGGCTCTGTTCAGAAAGCAGTACCCCCAGCACCGATCTAAGGAGATCGACCGATGCATCGCCAAGGCCATCCGATACATCGAGAACATGCAGAACCCCGACGGCTCTTGGTATGGATGCTGGGGCATCTGTTACACCTACGGCACCTGGTTCGCTGTGGAGGGCCTGACTGCTTGTGGAAAGAACTGCCACAACTCTCTGTCTCTGCGAAAGGCCTGCCAGTTCCTGCTGTCTAAGCAGCTGCCTAACGCCGGATGGGGCGAATCTTACCTGTCTTCTCAGAACAAGGTGTACACCAACCTGGAGGGCAACAGAGCTAACCTGGTGCAGTCTTCTTGGGCCCTGCTGTCTCTTACTCACGCTGGACAGGCCGAGATTGATCCCACTCCCATCCACAGAGGCATGAAGCTGCTGATCAACTCTCAGATGGAGGACGGCGATTTTCCCCAGCAGGAGATCACTGGCGTGTTCATGCGAAACTGCACCCTGAACTACTCTTCTTACCGAAACATCTTCCCCATCTGGGCCATGGGCGAGTACAGAAGACAAGTGCTGTGCGCCCACTCTTATTAA

*> OeLus* from *Olea europaea*

ATGTGGAAGCTGAAGATCGCCGATGGAACTGGACCTTGGCTGACCACTACCAACAACCACATCGGCCGACAGCACTGGGAATTCGATCCTGAAGCTGGCACTCCCGACGAAAGAGTGGAAGTGGAGAGACTGCGAGAGGAGTTCAAGAAGAACCGATTCCGAACCAAGCAGTCTGCCGACCTGCTGATGAGAATGCAGCTGGTGAAGGAGAACCAGCGAGTGCAAATTCCCCCCGCCATCAAGATTAAGGAAACCGAGGGCATTACTGAGGAGGCCGTGATCACTACTCTGCGACGAGCCATCTCTTTCTACTCTACCATCCAGGCTCATGACGGACATTGGCCTGCTGAATCTGCTGGCCCCCTGTTTTTTCTGCCTCCTCTGGTGCTGGCTCTGTATGTGACCGGAGCCATCAACGTGGTGCTGTCTCGAGAGCACCAGAAGGAGATCACCCGATACATCTACAACCACCAGAACGAGGATGGCGGCTGGGGAATTCATATCGAGGGCCACTCTACCATGTTCGGCTCTGTGCTGTCTTACATCACCCTGCGACTGCTGGGAGAAGGACAAGAGGACGGCGAAGATAAAGCTGTGGCCCGAGGCAGAAAGTGGATTCTGGATCACGGAGGCGCTGTGGGAATTCCTTCTTGGGGCAAGTTCTGGCTTACTGTGCTGGGCGTGTATGAATGGGATGGCTGCAACCCTATGCCTCCCGAATTTTGGCTGCTGCCCAACTTCTCTCCTATCCACCCCGGCAAAATGCTGTGCTACTGCCGACTGGTGTACATGCCCATGTCTTACCTGTACGGCAAGCGATTCGTGGGACCCATTACCGGACTGGTGCTGTCTCTGCGACAGGAGATCTACACCGAACCCTACCACGGCATCAATTGGAACCGAGCCCGAAACACCTGTGCTAAGGAGGACCTGTACTACCCTCATCCTCTGGCCCAAGATATGCTGTGGGGCTTCCTGCATCACTTCGCCGAACCTGTGCTTACTCGATGGCCCTTCTCTAAGCTGCGAGAGAAGGCCCTGAAAGTGGCCATGGAGCACGTTCACTACGAGGACATGAACTCTCGATACCTGTGCATCGGCTGCGTGGAGAAAGTGCTGTGCCTGATCGCTTGCTGGGTGGAAGATCCCAATTCTGAGGCCTACAAGCGACACATTGCCCGAATCCCCGACTACTTCTGGGTTGCTGAAGACGGCCTGAAGATGCAGTCTTTCGGCTGCCAGATGTGGGATGCTGCCTTCGCTATTCAGGCCATCCTGTCTTCTAACCTGGCCGAGGAGTATGGACCCACCCTGATGAAAGCCCACAACTTCGTGAAGGCCTCTCAGGTGCAGGAAAATCCCTCTGGCGACTTCAACGAGATGTACCGACACACCTCTAAAGGCGCCTGGACCTTCTCTATGCAGGACCACGGCTGGCAAGTGTCTGATTGCACCGCCGAGGGACTGAAAGCTGCCCTGCTGTTTTCTCAGATGCCCATCGAGCTGGTGGGAGCTGAAATCGAGACTGGCCACCTGTACGATGCCGTGAACGTGATCCTGACCCTGCAATCTGCCTCTGGCGGATTTCCTGCTTGGGAACCCCAAAAAGCCTACCGATGGCTGGAAAAGCTGAACCCCACCGAGTTCTTCGAGGACGTGCTGATCGAGCGAGACTACGTGGAGTGTACCTCTTCTGCTGTGCAAGCCCTGAAGCTGTTCAAACAGCTGCACCCCGGCCATCGAAGAAAAGAGATCGCCTCTTGCATCTCTAAGGCCATCCAGTACATCGAGGCCACTCAGAACCCCGATGGATCTTGGGACGGCTCTTGGGGAATCTGCTTCACCTACGGCACCTGGTTCGCTGTTGAAGGACTGGTGGCCTGTGGAAAGAACTACCACAACTCTCCCACCCTGAGAAGAGCTTGCGAGTTCCTGCTGTCTAAGCAGCTGCCTGACGGAGGATGGTCTGAGTCTTACCTGTCTTCTTCTAACAAGGTGTACACCAACCTGGAGGGCAACCGATCTAACCTGGTGCAGACCTCTTGGGCTCTGCTGTCTCTGATTAAGGCCGGCCAGGTGGAAATTGACCCTGGACCTATCCACAGAGGCATCAAGCTGCTGGTGAACTCTCAGATGGAGGACGGCGATTTCCCCCAGGAGGAAATCACTGGCGCCTTCATGAAGAACTGCACCCTGAACTACTCTTCTTACCGAAACATCTTCCCCATCTGGGCCCTGGGAGAGTATCGACGACGAATCCTGCATGCCCAGACTTAA

*> BgLus* from *Bruguiera gymnorhiza*

ATGTGGCGACTGAAGATCGCCGAAGGAGGCAACAACCCCTACATCTACTCTACCAACAACTTCGTGGGCCGACAGACCTGGGAATTCGACCCCGAAGCTGGAACTCCTGAGGAAAGAGCCCAAGTGGAAGAGGCTCGAGAGAACTTCTGGCGAGATCGATTCCTGATCAAGCCCTCTTCTGACCTGCTGTGGCGATTCCAGTTCCTGTCTGAGAAGAAGTTCAAGCAGCGAATCCCCCAGGTGAAAGTGCAAGACGGCGAGGAAATCACTCGAGAGATCGCCACTACCGCTCTGCGAAGATCTGTGCATCTGGTGTCTGCCCTGCAAGCTTCTGACGGACATTGGTGCGCCGAGAATTCTGGCCCCATGTTCTTTGTTCCCCCCATGGTGTTCTCTCTGTACATCACCGGCCACCTGAACGCTGTTTTCTCTGCCGAGCATTGCAAGGAGATCCTGCGATACATCTACTGCCACCCCAACGAAGATGGAGGATGGGGCCTGCACATTGAAGGACACTCTGCCATGTTCTCTACCGTGCTGAACTACAACTGGCTGGGCAAACTGGGCGAAGGACGAGATGGAGGCAAAGATAACGCCTGCGAACGAGCCAGACGACGAATTCTGGATCACGGATCTGCCACCGCCATTTCTTCTTGGGGCAAGACCTGGCTTGCTATTCTGGGCGTGTATGAGTGGGATGGCTGTAATCCTATGCCCCCCGAATTCTGGGCTTTCCCCACCTTCTTTCCTATTCACCCCGCCCGAATGCTTTGTTACTGCCGACTGACCTACATGGCCATGTCTTACCTGTACGGCAAGAAGTTCGTGGGACCCATCACCCCTCTGATCCTGCAACTGCGAGAGGAGATCTACAACGAGCCCTACGACCAGATCAACTGGTCCCGAATGCGACACCTGTGTGCCAAGGAGGACAACTACTACGCCCACACCCTGACCCAGATCATTCTGTGGGACGCCATCTATATGCTGGGAGAGCCCCTGCTGAAAAGATGGCCCTTCAACAAGCTGCGAGAGAAGGCCCTGAAGATCACCATGGACCACATCCACTACGAGGACGAGAACTCTCAGTACATCACCATCGGCTCTGTGGAGAAGCCTCTGCTGATGCTGGCTTGTTGGCACGAGGACCCTAATGGAGATGCCTTTAAGAAGCACCTGGCCCGAATCCCTGACTATGTGTGGCTGGGAGAAGACGGCATCAAGATCCAGTCTTTCGGCTCTCAGGTGTGGGACACCTCTTTCGTGCTGCAAGCCCTGATCGCTTCTAACCTGCCCTCTGAAACTGGCCCCACTCTGGAAAAAGGCCACAACTTCATCAAGAACTCTCAGGTGACCCAGAACCCTTCTGGCGACTTCCGACGAATGTTCCGACACATCTCTAAGGGCTCTTGGACCTTCTCTGACAAGGACCACGGCTGGCAGGTGTCTGATTGCACAGCCGAGTCTCTGAAGTGCTGCCTGCTGTTCTCTATGATGCCCCCCGAGCTGGTGGGAGAAAAGATGGGACCCCAGCGAATGTATGACGCCGTGAACGTGATCATCTCTCTGCAGTCTAAGAACGGCGGATGCTCTGCTTGGGAACCTGCTGGAGCTGGCTCTTGGATGGAATGGCTGAACCCCGTGGAATTCCTGGCTGACCTGGTGATCGAACACGAGTACGTGGAGTGTACCTCTTCTTCTCTGCAGGCCCTGGTCTGTTCAAGAAGCTGTACCCCGAGCACCGACGAAAGGAGATCGAGATCTTCATCCTGAACGCCGTGCGATTCACCGAGGAAATCCAGCAGCCTGACGGATCTTGGTACGGCAATTGGGGCATCTGCTTCCTTTCTGGCACCTGGTTTGGCCTGAAAGGACTGGCTGCTGCCGGAAAGACCTACTACAACTGCACCGCCGTGAGAAAGGGAGTGGAGTTCCTGCTGCAAACCCAGCGAGATGATGGAGGATGGGGAGAGTCTTACCTGTCTTGCCCCAAGAAGATCTACGTGCCCCTGGAGGGCAACAGATCTAACCTGGTGCAGACCGCCCTTGCTATGATGGGACTGATCCTGGGAGGACAGGGCGAAAGAGATCCTACCCCCCTGCATAGAGCTGCCAAGCTGCTGATCAACTCTCAGACCGAGCTGGGAGATTTTCCCCAGCAGGAACTGTCTGGCTGCTTCATGCGAAACTGCATGCTGCACTACTCTGAGTACCGAGACATCTTTCCCACTTGGGCCCTGGCCGAATACTGTAAGCTGTTCCCCCTGCCCTCTAAGAATGACTAA

*> KdLus* from *Kalanchoe daigremontiana*

ATGTGGAAGCTGAAGATCGCCGACGGAGGCTCTAACCCCTACATCTTCACCACCAACAACTTCGTGGGCCGACAGATCTGGGAGTTCGATCCCCAAGCCACTGATCCCCAGCAACTGGCTAAAGTGGAAGCTGCCCGACTGGACTTTTACCACAACCGATACAAGCTGAAGCCCAACTCTGACCTGCTGTGGCGAATGCAGTTCCTGGAGGAGAAGGCCTTCACCCAGACCATTCCCCAGGTGAAGGTGGAAGACGGCGAGGAAGTGTCTTACGAGGCTGTGACTGCTGCTCTCAGACGAGGAGTCCACCTGTATTCTGCTCTGCAGGCCTCTGATGGACATTGGCCTGCTGAGAATGCCGGCCCCATGTTTTTTATGCCCCCCATGGTGATGTGCCTGTACATCACCGGCCACCTGAACGCCATTTTCACCGAGGAGCACCGATCTGAAACCCTGCGATACATCTACTACCACCAGAACGAGGATGGAGGCTGGGGCTTTCATATCGAAGGCCACTCTACCATGTTCGGCACCGTGCTGAACTACATCTGCATGCGACTGCTGGGAGAAGGACCTGAGGGAGGACAGGATAATGCCGTGTCTCGAGGCCGAAAGTGGATTCTGGATCATGGCGGCGCCACCTCTATTCCTTCTTGGGGCAAGACCTGGCTGTCTATCATGGGCCTGTGCGATTGGTCTGGCTGCAATCCTATGCCCCCCGAGTTTTGGCTGCTGCCTTCTTACCTGCCTATGCACCCCGGAAAAATGTGGTGCTACTGCCGAATGGTGTACATGCCCATGTCTTACCTGTACGGCAAGCGATTCACCGCTCGAATTACCCCCCTGATCCTGCAACTGCGAGAGGAGATCCACATCCAGCCCTACGACCAGATCGACTGGAAGAAGGTGCGACACGTGTGCTGCAAGGAGGACATGTACTACCCCCACCCTCTGCTGCAAGATCTGCTGTGGGACACCCTGTATCTGACCACCGAGCCTCTGCTTACTAGATGGCCCCTGAACAAGCTGATCCGAAAGCGAGCTCTGCAGACCACCATGAAGCACATCCACTACGAGGACGAGAACTCTCGATACATCACCATCGGCTGCGTGGAGAAGGTGCTGTGTATGCTGGCCTGTTGGGTGGAAGATCCCAATGGCGACTACTTCAAGAAGCACCTGGCCCGAATCCCTGATTACCTGTGGATTGCCGAGGACGGCATGAAGATGCAGTCTTTCGGCTCTCAGCATTGGGACACCGCCTTCTCTATTCAAGCCCTGCTGGCCTCTAACATGGCTGAGGAGATCGGCATCACCCTGGCTAAAGGCCACGACTTCATCAAGAAGTCTCAGGTGAAGGACAACCCCTCTGGCGACTTCAAGGGCATGTACCGACACATCTCTAAGGGCGCCTGGACCTTCTCTGATCAGGATCACGGCTGGCAGGTGTCTGATTGTACCGCCGAAGGACTGAAATGCTGCCTGCTGTTCTCTATGATGCAGCCCGAGGTGGTGGGAGAATCTATGGCCCCTGAGTCTCTGTACAACTCTGTGAACGTGCTGCTGTCTCTGCAGTCTCAGAACGGAGGACTGCCTGCTTGGGAACCTGCTGGAGCTCCCGAATGGCTTGAACTGCTGAATCCCACCGAGTTCTTCGAGAACATCGTGATCGAGCACGAGTACGTGGAGTGTACCTCTTCTGCTGTGCAAGCCCTGGTGCTGTTCAAGAAGCTGTACCCCCTGCACCGAAGAAAAGAGGTGGAGCGATTCATTACCAACGGCGCCAAGTACCTGGAGGACATCCAAATGCCCGACGGCTCTTGGTATGGAAATTGGGGCGTGTGCTTCACTTACGGCGCTTGGTTTGCTCTGGAGGGACTGTCTGCTGCTGGCAAGACCTATAACAACTGCGCCGCCGTGAGAAAGGGAGTGGACTTCCTGCTGAACATCCAGCTGGAAGACGGAGGATGGGGAGAGTCTTACCAGTCTTGCCCCGACAAGAAGTATGTGCCCCTGGAGGACAACCGATCTAACCTGGTGCAGACCTCTTGGGCTCTGATGGGCCTGATCTATGCTGGCCAAGCCGATCGAGATCCCACTCCTCTGCACAGAGCTGCTCAGCTGCTGATTAACTCTCAGCTGGAGGACGGCGATTTTCCCCAGCAAGAGATCACTGGCGTGTTCCAGCGAAACTGCATGCTTCACTACGCCGCCTACCGAAACATCTTTCCCCTGTGGGCCCTGGCCGAATACAGACGACAGATCCAGCTGCATTCTGAGGCCACCAAGATGGTTTAA

*> RcLus* from *Ricinus communis*

ATGTGGCGAATCAAGATCGCCGAGGGCGGAAACAACCCCTACATCTACTCTACCAACAACTTCCAGGGCCGACAGATCTGGGTGTTTGACCCCAATGCCGGAACTCCCGAAGAACAAGCCGAAGTGGAAGAAGCCAGACAGAACTTCTGGAAGAACCGATTCCAGGTGAAGCCCAACTCTGACCTGCTGTGGCAGCTGCAATTCCTGCGAGAGAAGAACTTCAAGCAGAAGATCCCCAAGGTGAAGGTGGAAGACGGCGAGGAAATCACCTCTGAGATTGCTGCTGCCGCTCTGAGAAGATCTGTGCATCTGTTCTCTGCTCTGCAGGCCTCTGATGGACATTGGTGCGCTGAAAATGGCGGCCTGCTGTTTTTTCTTCCCCCCCTGGTGTTTGCTGTGTACATCACCGGACACCTGAACACCGTGTTCTCTCCCGAGCACCGAAAGGAGATCCTGCGATACATCTACTGCCACCAGAATGAAGATGGCGGCTGGGGAATTCATATCGAGGGCCACTCTACCATGTTCTGCACCGTGCTGAACTACATCTGCATGCGAATCCTGGGCGAAGCTAGAGATGGCGGAATTGAGAACGCTTGTGAGCGAGGCAGAAAGTGGATTCTGGACCATGGAGGAGCCACCGGAATTTCTTCTTGGGGCAAGACCTGGCTTTCTATCCTGGGCGTGTACGAATGGGACGGAACCAACCCTATGCCTCCCGAGTTTTGGGCTTTCCCCTCTTCTTTCCCTCTGCACCCCGCTAAGATGTTCTGCTACTGCCGAATCACCTACATGCCCATGTCTTACCTGTACGGCAAGAGATTCGTGGGCCCCATCACTCCTCTGATCCTGCAAATCCGAGAGGAGATCTACAACGAGCCCTACAACAAGATCAAGTGGAACTCTGTGCGACACCTGTGCGCTAAGGAGGACAACTATTTCCCCCACCCCACCATCCAAAAGCTGCTGTGGGATGCCCTGTACACCTTTTCTGAGCCCCTGTTCTCTCGATGGCCCTTCAACAAGCTGCGAGAGAAGGCCCTGAAGATCACCATGGACCACATCCACTACGAGGACCACAACTCTCGATACATCACCATCGGCTGCGTGGAAAAGCCCCTGTGCATGCTGGCTTGTTGGATCGAGGACCCCCATGGCGAGGCTTTCAAAAAGCACCTGGCCCGAATCGCCGACTATATCTGGGTTGGAGAGGACGGCATCAAGATGCAGTCTTTCGGCTCTCAGACCTGGGATACCTCTCTGGCTCTGCAAGCCCTGATTGCCTCTGATCTGTCTCACGAGATCGGACCCACTCTGAAACAGGGCCACGTGTTCACCAAGAACTCTCAGGCCACCGAGAATCCTTCTGGCGACTTCCGAAAGATGTTCCGACACATCTCTAAGGGCGCCTGGACCTTCTCTGATAAGGACCAGGGCTGGCAAGTGTCTGATTGCACCGCCGAGTCTCTGAAATGCTGCCTGCTGTTCTCTATGATGCCCCCCGAGATCGTGGGAGAAAAGATGGAGCCCGAGAAGGTGTACGACTCTGTGAACGTGATCCTGTCTCTGCAGTCTCAGAACGGAGGATTCACAGCCTGGGAACCTGCTAGAGCTGGCTCTTGGATGGAATGGCTGAACCCCGTGGAGTTCATGGAAGACCTGGTGGTGGAGCACGAGTATGTGGAGTGTACCTCTTCTGCCATCCAAGCCCTGGTGCTGTTCAAGAAGCTGTACCCCCGACACCGAAACAAGGAGATCGAGAACTGCATCATCAACGCCGCCCAGTTCATCGAGAACATCCAGGAGCCTGACGGCTCTTGGTATGGCAATTGGGGCATCTGCTTCTCTTACGGCACCTGGTTTGCCCTTAAAGGCCTGGCTGCTGCTGGAAGAACCTACGAGAACTGCTCTGCCATCCGAAAGGGCGTTGACTTCCTGCTGAAGTCTCAGAGAGATGATGGCGGCTGGGCTGAATCTTACCTGTCTTGCCCCAAGAAGGTGTACGTGCCCTTCGAGGGCAACCGATCTAACCTGGTGCAGACCGCTTGGGCTATGATGGGACTGATCTACGGCGGACAGGCCAAAAGAGATCCCATGCCCCTGCATAGAGCTGCCAAGCTGCTGATCAACTCTCAGACCGACCTGGGCGATTTCCCTCAGCAAGAGCTGACTGGCGCTTTCATGCGAAACTGCATGCTGCACTACGCCCTGTTCCGAAACACCTTCCCCATTTGGGCTCTGGCCGAATATCGACGACACGTGCTGTTTCCTTCTGCCGGATTCGGATTCGGCTTCACCAACAACCTGTAA

*> αAS* from *Malus domestica*

ATGTGGAAGATCAAGTTCGGCGAGGGAGCCAACGATCCCATGCTGTTCTCTACCAACAACTTCCACGGCCGACAGACTTGGGAGTTCGATCCTGATGCCGGCACTGAAGAGGAAAGAGCTGAGGTGGAAGCTGCTCGAGAGCACTTCTACCAGAACCGATTCAAGGTGCAGCCCTCTTCTGATCTGCTGTGGCGATTCCAGATCCTGCGAGAGAAGAACTTCAAGCAGGAGATCCCCCCTGTTAGAGTGGGAGAGGGCGAGGATATCACCTACGATCAGGCTACTGCCGCTTTTCGACGAGCTGCTACCTTTTGGAACGCCCTGCAGTCTCCTCATGGACACTGGCCTGCTGAAAATGCCGGCCCCAACTTTTATTTTCCCCCCCTGGTGATGGCTGTTACATTCCCGGCTACCTGAACGTGATCTTCTCTGCCGAGCACAAGAAGGAGATCCTGCGATACACCTACAACCACCAGAACGAGGATGGAGGATGGGGACTGCATATTGCTGGCCCCTCTATGATGTTCACCACCTGCCTGAACTACTGCATGATGCGAATCCTGGGAGATGGACCCGATGGAGGACGAGACAACGCTTGTGCTCGAGCCCGAAAGTGGATTCTTGATCGAGGCGGCGCTTACTATTCTGCCTCTTGGGGCAAGACTTGGATGGCTATCCTGGGCGTGTATGACTGGGAAGGCTCTAATCCTATGCCCCCCGAATTTTGGACTGGCTCTACCCTTCTTCCCTTCCACCCCTCTAAGATGTTCTGCTACTGCCGACTGACCTACCTGCCCATGTCTTACTTCTACGCCACCCGATTCGTGGGACCTATCACTCCTCTGGTGGAGGAACTGCGACAGGAGATCTACTGCGAGTCTTACAACGAGATCAACTGGCCCAAGGTGCGACATTGGTGCGCTACCGAGGACAACTATTACCCCCACGGCAGAGTGCAACGATTCATGTGGGACGGCTTCTACAACATCGTGGAGCCCCTGCTTAAACGATGGCCCTTCAAGAAGATCCGAGACAACGCCATCCAGTTCACCATCGACCAGATCCACTACGAGGACGAGAACTCTCGATACATCACCATCGGCTGCGTGGAAAAGCCCCTGATGATGCTTGCTTGCTGGGCCGAAGATCCTTCTGGAGAGGCCTTCAAAAAACACCTGCCCCGAGTGACTGACTATATCTGGCTGGGCGAGGACGGAATCAAGATGCAGTCTTTCGGCTCTCAGTCTTGGGATTGCGCCCTGGTGATTCAAGCTCTGCTGGCCGGCAATCTGAACGCTGAAATGGGCCCCACCCTGAAAAAGGCTCACGAGTTCCTGAAGATCTCTCAGGTGCGAATCAACACCTCTGGCGACTACCTGTCTCACTTCCGACACATCTCTAAGGGCGCCTGGACCTTCTCTGATCGAGATCACGGCTGGCAAGTGTCTGATTGTACCGCCGAGGCTCTGAGATGCTGCTGCATCTTCGCCAACATGTCTCCCGAGGTGGTGGGAGAACCTATGGAAGCCGAGTGTATGTACGACGCCGTGAACGTGATCATGTCTCTGCAGTCTCCCAATGGAGGCGTGTCTGCTTGGGAACCTACTGGAGCCCCCAAATGGCTTGAGTGGCTGAACCCTGTGGAGTTCCTGGAAGACCTGGTGATCGAGTACGAGTACATCGAATGCACCTCTTCTTCTATCCAGGCCCTGACCCTGTTCAGAAAGCTGTACCCCGGCCATCGACGAAAGGAGATCAACAACTTCATCACCCGAGCCGCCGACTATATCGAGGACATCCAGTACCCCGACGGATCTTGGTATGGCAATTGGGGCATCTGCTTCGTTTACGGCACCTGGTTCGCCATTAAAGGACTGGAAGCCGCTGGCCGAACCTATAATAACTGCGAGGCCGTGAGAAAAGGCGTGGACTTCCTGCTGAAGACCCAGAGAGCTGATGGCGGATGGGGAGAACACTACACCTCTTGCACCAACAAGAAGTACACCGCCCAGGACTCTACTAACCTGGTGCAGACCGCTCTTGGACTGATGGGACTGATCCATGGCAGACAGGCTGAGAGAGATCCTACTCCCATCCACAGAGCTGCTGCTGTGCTGATGAATGGACAGCTGGACGATGGAGATTTCCCCCAGCAAGAGCTGATGGGCGTGTTCATGCGAAACGCCATGCTGCACTACGCTGCCTACCGAAACATCTTCCCCCTGTGGGCTCTGGGAGAATACCGAACCCTGGTGTCTCTGCCCATCAAGAAGATCGCCTAA

*> βAS* from *Arabidopsis thaliana*

ATGTGGCGACTGAAGATCGGCGAAGGAAACGGCGATGACCCCTATCTGTTCACCACCAACAACTTCGCCGGCAGACAGACCTGGGAGTTCGATCCTGATGGCGGCTCTCCTGAGGAAAGACACTCTGTGGTGGAGGCCCGACGAATCTTCTACGACAACCGATTCCACGTGAAGGCCTCTTCTGACCTGCTGTGGCGAATGCAGTTCCTGCGAGAGAAGAAGTTCGAGCAGCGAATCGCTCCTGTGAAGGTGGAGGACTCTGAGAAGGTGACCTTCGAAACCGCTACCTCTGCTCTGCGAAGAGGCATCCACTTCTTCTCTGCCCTGCAAGCTTCTGATGGCCATTGGCCTGCTGAAAATGCTGGCCCTCTGTTTTTTCTGCCTCCCCTGGTGTTCTGCCTGTACATCACCGGCCACCTGGATGAGGTGTTCACCTCTGAGCACCGAAAGGAGATCCTGCGATACATCTACTGCCACCAGAAGGAAGATGGAGGCTGGGGACTGCATATTGAGGGCCACTCTACCATGTTCTGCACCACCCTGAACTACATCTGCATGCGAATCCTGGGAGAGTCTCCCGATGGCGGACATGATAATGCTTGCGGCCGAGCTAGAGAGTGGATTCTGTCTCATGGCGGCGTGACCTACATTCCCTCTTGGGGCAAGACCTGGCTGTCTATCCTGGGCGTGTTCGACTGGTCTGGCTCTAATCCTATGCCCCCCGAGTTTTGGATTCTGCCCTCTTTCTTTCCTGTCCACCCCGCCAAGATGTGGTCTTACTGCCGAATGGTGTACCTGCCCATGTCTTACCTGTACGGCAAGAGATTCGTGGGCCCCATCACCTCTCTGATCCTGCAGCTGCGAAAGGAGCTTTACCTGCAGCCCTACGAGGAGATCAACTGGATGAAGGTGCGACACCTGTGCGCTAAAGAGGACACCTACTACCCTAGACCCCTGGTGCAAGAACTGGTGTGGGACTCTCTGTACATCTTCGCCGAGCCCTTCCTTGCTAGATGGCCCTTCAACAAGCTGCTGCGAGAGAAGGCTCTGCAACTGGCCATGAAGCACATCCACTACGAGGACGAGAACTCTCGATACATCACCATCGGCTGCGTGGAAAAGGTGCTGTGCATGCTGGCTTGTTGGGTGGAAGATCCCAACGGCGACTACTTCAAGAAGCACCTGTCTCGAATCTCTGACTACCTGTGGATGGCTGAGGACGGCATGAAGATGCAGTCTTTCGGCTCTCAGCTGTGGGATACCGGCTTCGCTATGCAAGCTCTGCTGGCCTCTAACCTGTCTTCTGAGATCTCTGACGTGCTCAGACGAGGCCACGAGTTCATCAAGAACTCTCAGGTGGGCGAGAATCCTTCTGGCGACTACAAGTCTATGTACCGACACATCTCTAAGGGCGCCTGGACCTTCTCTGATCGGGACCACGGCTGGCAAGTGTCTGACTGTACTGCTCACGGCCTGAAGTGTTGCCTGCTGTTCTCTATGCTGGCCCCCGATATTGTGGGACCCAAGCAGGACCCTGAACGACTGCATGACTCTGTGAACATCCTGCTGTCTCTGCAGTCTAAGAACGGCGGCATGACTGCTTGGGAACCTGCTGGAGCTCCTAAATGGCTGGAACTGCTGAATCCCACCGAGATGTTCTCTGACATCGTGATCGAGCACGAGTACTCTGAGTGTACCTCTTCTGCTATCCAGGCCCTGTCTCTGTTCAAGCAGCTGTACCCCGACCATCGAACCACTGAGATCACCGCCTTCATCAAGAAGGCCGCCGAGTACCTGGAAAACATGCAGACCCGAGATGGCTCTTGGTATGGCAATTGGGGCATCTGCTTCACTTACGGCACCTGGTTTGCTCTGGCTGGACTGGCTGCTGCTGGAAAGACCTTCAATGACTGCGAGGCCATCAGAAAGGGCGTGCAATTTCTTCTGGCCGCCCAGAAAGATAATGGAGGCTGGGGCGAATCTTACCTGTCTTGCTCTAAGAAGATCTACATCGCCCAGGTGGGCGAGATTTCTAACGTGGTGCAGACTGCCTGGGCTCTGATGGGACTGATCCACTCTGGCCAGGCTGAACGAGATCCCATTCCTCTTCACAGAGCCGCCAAGCTGATCATCAACTCTCAGCTGGAGTCTGGCGATTTCCCTCAACAGCAGGCCACTGGAGTGTTCCTGAAGAACTGCACCCTGCACTATGCCGCCTACCGAAACATTCATCCCCTGTGGGCTCTGGCTGAGTATCGAGCCAGAGTGTCTCTTCCTTAA

*> TPS* from *Pinus sylvestris*

ATGGCTCAGATTTCCATCGGTGCCCCCCTGTCCGCCGAGGTCAACGGTGCTTGCATCAACACCCACCACCACGGTAACCTGTGGGACGACTACTTCATCCAGTCCCTGAAGTCTCCCTACGAGGCCCCCGAGTGCCACGAGCGATGCGAGAAGATGATCGAGGAGGTGAAGCACCTGCTGCTGTCCGAGATGCGAGACGGTAACGACGACCTGATTAAGCGACTGCAGATGGTCGACATCTTCGAGTGTCTGGGTATTGACCGACACTTCCACCACGAGATCCAGGCCGCCCTGGACTACGTCTACCGATACTGGAACGAGCTGGAGGGCATCGGCGTCGGCACCCGAGACTCTCTGACCAAGGACCTGTACGCCACCGGCCTGGGTTTCCGAGCCCTGCGACTGCACCGATACAACGTCTCTTCTGCCGTCCTGGAGAACTTCAAGAACGAGAACGGTCTGTTCTTCCACTCTTCCGCCGTGCAGGAGGAGGAGGTCCGATGCATGCTGACCCTGCTGCGAGCCTCTGAGATCTCCTTCCCCGGTGAGAAGGTCATGGACGAGGCCAAGGCCTTCGCCACCGAGTACCTGAACCAGCTGCTGACCCGAGTGGACATTACCGAGGTCGGCGAGAACCTGCTGCGAGAGGTGCGATACGCCCTGGACTTCCCCTGGTACTGTTCTGTCCCCCGATGGGAGGCCCGATCCTTCATCGAGATTTTCGGCCAGAACAACTCTTGGCTGAAGTCCACCATGAACAAGAAGGTGCTGGAGCTGGCCAAGCTGGACTTCAACATTCTGCAGTCCGCCCACCAGCGAGAGCTGCAGCTGCTGTCCCGATGGTGGTCCCAATCTGACATTGAGAAGCAGAACTTCTACCGAAAGCGACACGTCGAGTTCTACTTCTGGATGGTCATCGGTACTTTCGAGCCCGAGTTCTCCTCCTCCCGAATTGCCTTCGCCAAGATCGCCACCCTGATGACCATCCTGGACGACCTGTACGACACCCACGGTACTCTGGAGCAGCTGAAGATTTTCACCGAGGCCGTCAAGCGATGGGACCTGTCCCTGCAGGACCGACTGCCCGACTACATTAAGATCACCCTGGAGTTCTTCTTCAACACCTCCAACGAGCTGAACGCCGAGGTGGCCAAGATGCAGGAGCGAGACATGTCCGCCTACATCCGAAAGGCCGGCTGGGAGCGATACATTGAGGGTTACATGCAGGAGTCCGAGTGGATGGCCGCCCGACACGTGCCCACCTTCGACGACTACATGAAGAACGGCAAGCGATCCTCTGGCATGTGTATTCTGAACCTGTACTCCCTGCTGCTGATGGGCCAGCTGGTCCCCGACAACATCCTGGAGCAGATCCACCTGCCCTCCAAGATCCACGAGCTGGTCGAGCTGACCGCCCGACTGGTCGACGACTCCAAGGACTTCCAGGCCAAGAAGGACGGCGGTGAGTTCGCCTCTGGCACCGAGTGCTACCTGAAGGAGAAGCCCGAGTGTACTGAGGAGGACGCCATGAACCACCTGATTGGCCTGCTGAACCTGACCGCCATGGAGCTGAACTGGGAGTTCGTGAAGCACGACGGCGTCGCCCTGTGTCTGAAGAAGTTCGTCTTCGAGGTCGCCCGAGGCCTGCGATTCATCTACAAGTACCGAGACGGTTTCGACTACTCCAACGAGGAGATGAAGTCCCAGATCACCAAGATTCTGATCGACCAGGTGCCCATTTAA

Table S2. Subcellular targeting signal peptides used in this study.

| Location | Amino acid sequence | Signal peptide | Source |
| --- | --- | --- | --- |
| Mit | MLSLRQSIRFFKRSGI | N-terminus | [1] |
| Per | SKL | N-terminus | [2] |
| ER | KDEL | N-terminus | [2] |

**Table S3. Upregulated genes in the membrane phospholipid synthesis pathway.**

| Genes | | YALI no. | Reference |
| --- | --- | --- | --- |
| *SCT1* | *YALI0_C00209g* | | This work |
| *SLC1* | *YALI0_E18964g* | | This work |
| *CHO1* | *YALI0_D08514g* | | This work |
| *PSD1* | *YALI0_D21604g* | | This work |
| *OPI3* | *YALI0_E12441g* | | This work |

**Table S4.** Lupeol yield and productivity in selected strains.

| Strains | Lupeol yield (mg/L) | Lupeol productivity (mg/L/OD/d) |
| --- | --- | --- |
| LU-9 | 29.00±1.03 | 0.22±0.10 |
| LU-20 | 91.74±5.74 | 0.40±0.01 |
| LU-21 | 95.66±6.44 | 0.49±0.05 |
| LU-22 | 137.52±1.48 | 0.56±0.07 |

**Table S5. Strains used in this work.**

| Strains | Relative characteristics | Source |
| --- | --- | --- |
| *Yarrowia lipolytica* ATCC 201249 | *MATA, ura3-302, leu2-270, lys8-11, PEX17-HA, Ref* | [3] |
| LU-1 | ATCC 201249 harboring pINA1269-*AtLus* | This work |
| LU-2 | ATCC 201249 harboring pINA1269-*GuLus* | This work |
| LU-3 | ATCC 201249 harboring pINA1269-*OeLus* | This work |
| LU-4 | ATCC 201249 harboring pINA1269-*BgLus* | This work |
| LU-5 | ATCC 201249 harboring pINA1269-*KdLus* | This work |
| LU-6 | ATCC 201249 harboring pINA1269-*RcLus* | Initial strain |
| LU-7 | ATCC 201249 Δ*ku80*:: *P_FBAin_*-*RcLus*-*octt*, hisG | This work |
| LU-8 | ATCC 201249 Δ*ku80*:: *P_Hp8d_*-*RcLus*-*fba1t*, hisG | This work |
| LU-9 | ATCC 201249 Δ*ku70*:: *P_TEFin_*-*RcLus*-*acot*, hisG | Control strain |
| LU-10 | LU-9 rDNA:: *P_TEFin_-HMG1-octt*, Hph | This work |
| LU-11 | LU-9 rDNA:: *P_EXP1_-ERG1-xpr2t*, *P_TEFin_-ERG9-lip2t*, *P_TEFin_-HMG1-octt*, Hph | This work |
| LU-12 | LU-9 *pxa1-532* | This work |
| LU-13 | LU-9 *MGA2 1927G>C* | This work |
| LU-14 | LU-9 *pex10-1563*, *mfe1-541* | This work |
| LU-15 | LU-9 *pot1-634* | This work |
| LU-16 | LU-9 *lro1-3* | This work |
| LU-17 | LU-9 *rpd3-447* | This work |
| LU-18 | LU-9 IntE:: *P_TEFin_*-*ACC1*-*pex20t*, hisG | This work |
| LU-19 | LU-9 Δ*snf1*:: hisG | This work |
| LU-20 | LU-9 IntE:: *P_TEFin_*-*OLE1*-*pex20t*, hisG | This work |
| LU-21 | LU-9 *pah1-514*, *dgk1-505* | This work |
| LU-22 | LU-21 IntE:: P*TEFin*-*OLE1-pex20t*, hisG | This work |
| LU-23 | LU-11 *pah1-514*, *dgk1-505,* Δ*ku80*:: *P_FBAin_*-*OLE1*-*octt*, hisG | This work |
| LU-28 | ATCC201249 IntE:: *P_TEFin_-OLE1-pex20t*, hisG | This work |
| LU-29 | ATCC201249 *pah1-514, dgk1-505* | This work |
| LU-30 | ATCC201249 *pah1-514,* *dgk1-505, IntE*:: *P_TEFin_-OLE1-pex20t*, hisG | This work |
| GFP-1 | ATCC 201249 Δ*ku70*:: *P_TEFin_*-*GFP*-*acott*, hisG | This work |
| GFP-2 | ATCC 201249 Δ*ku70*:: *P_TEFin_*-*GFP-*Mit-*acott*, hisG | This work |
| GFP-3 | ATCC 201249 Δ*ku70*:: *P_TEFin_*-*GFP-*Per-*acott*, hisG | This work |
| GFP-4 | ATCC 201249 Δ*ku70*:: *P_TEFin_*-*GFP-*ER-*acott*, hisG | This work |
| LU-31 | ATCC 201249 Δ*ku70*:: *P_TEFin_*-*RcLus-*Mit-*acott*, hisG | This work |
| LU-32 | ATCC 201249 Δ*ku70*:: *P_TEFin_*-*RcLus-*Per-*acott*, hisG | This work |
| LU-33 | ATCC 201249 Δ*ku70*:: *P_TEFin_*-*RcLus-*ER-*acott*, hisG | This work |
| aA-1 | ATCC 201249 *Δku70*:: P*TEFin*-αAS-acot, hisG | This work |
| aA-2 | aA-1 *Δku80*:: PFBAin-OLE1-octt, hisG | This work |
| aA-3 | aA-1 pah1-514, dgk1-505 | This work |
| aA-4 | aA-1 pah1-514, dgk1-505, *Δku80*:: PFBAin-OLE1-octt, hisG | This work |
| bA-1 | ATCC 201249 *Δku70*:: PTEFin-βAS-acot, hisG | This work |
| bA-2 | bA-1 *Δku80*:: PFBAin-OLE1-octt, hisG | This work |
| bA-3 | bA-1 *pah1-514*, *dgk1-505* | This work |
| bA-4 | bA-1 *pah1-514*, *dgk1-505,* Δ*ku80*:: *P_FBAin_*-*OLE1*-*octt*, hisG | This work |
| LO-1 | ATCC 201249 Δ*ku70*:: *P_TEFin_*-*TPS*-*acot*, hisG | This work |
| LO-2 | LO-1 Δ*ku80*:: *P_FBAin_*-*OLE1*-*octt*, hisG | This work |
| LO-3 | LO-1 *pah1-514*, *dgk1-505* | This work |
| LO-4 | LO-1 *pah1-514*, *dgk1-505,* Δ*ku80*:: *P_FBAin_*-*OLE1*-*octt*, hisG | This work |

**Table S6**. Plasmids used in this work.

| Plasmids | Relative characteristics | Source |
| --- | --- | --- |
| PINA 1269 | *Y. lipolytica* integrative plasmid, hp4d promoter, XPR2 terminator, LEU2 selection marker, Amp^R^ | [4] |
| PUC57-IntE-HUM | IntEup-*P_TEFin_*-*pex20t*-hisG-URA3-hisG-IntEdn cassette in PUC57 | This work |
| pINA1269-*AtLus* | Codon-optimizes *AtLus* gene was cloned into the *BamH*I/*Kpn*I site of pINA1269 | This work |
| pINA1269-*GuLus* | Codon-optimizes *GuLus* gene was cloned into the *BamH*I/*Kpn*I site of pINA1269 | This work |
| pINA1269-*OeLus* | Codon-optimizes *OeLus* gene was cloned into the *BamH*I/*Kpn*I site of pINA1269 | This work |
| pINA1269-*BgLus* | Codon-optimizes *BgLus* gene was cloned into the *BamH*I/*Kpn*I site of pINA1269 | This work |
| pINA1269-*KdLus* | Codon-optimizes *KdLus* gene was cloned into the *BamH*I/*Kpn*I site of pINA1269 | This work |
| pINA1269-*RcLus* | Codon-optimizes *RcLus* gene was cloned into the *BamH*I/*Kpn*I site of pINA1269 | This work |
| K8FB-*RcLus* | *P_FBAin_*-*RcLus*-*octt* cassette in PUC57-K8Zero-HUM | This work |
| Rs8d-*RcLus* | *RcLus* cassette in PUC57-RS8d-HUM | This work |
| IntK-*RcLus* | *RcLus* cassette in PUC57-IntK-HUM | This work |
| PUC57-IntK-*GFP* | *GFP* cassette in PUC57-RS8d-HUM | This work |
| PUC57-IntK- *GFP-Mit* | *GFP-Mit* cassette in PUC57-RS8d-HUM | This work |
| PUC57-IntK- *GFP-Per* | *GFP-Per* cassette in PUC57-RS8d-HUM | This work |
| PUC57-IntK- *GFP-ER* | *GFP-ER* cassette in PUC57-RS8d-HUM | This work |
| PUC57-IntK-*RcLus-Mit* | *RcLus-Mit* cassette in PUC57-RS8d-HUM | This work |
| PUC57-IntK-*RcLus-Per* | *RcLus-Per* cassette in PUC57-RS8d-HUM | This work |
| PUC57-IntK-*RcLus-ER* | *RcLus-ER* cassette in PUC57-RS8d-HUM | This work |
| rDNA-L-Hph-H0 | rDNAup-Hph-H0 | [5] |
| Yl-LD01 | H0-*P_EXP1_*-*xpr2t*-H1 | [5] |
| Yl-LD01-*ERG1* | H0-*P_EXP1_-ERG1-xpr2t*-H1 | [5] |
| Yl-LD02in | H1-*P_TEFin_-lip2t*-H2 | [5] |
| Yl-LD02in-*ERG9* | H1-*P_TEFin_-ERG9-lip2t*-H2 | [5] |
| Yl-LD03in | H2-*P_TEFin_-octt*-H3 | [5] |
| Yl-LD03in-*HMG1* | H2-*P_TEFin_-HMG1-octt*-H3 | [5] |
| H3-rDNA-R | H3-rDNAdn | [5] |
| PUC57-IntE-HUM | IntEup-*P_TEFin_*–*pex20t*-hisG-URA3-hiaG-IntEdn cassette in PUC57 | This work |
| PUC57-K8FB-*HUM* | Ku80up-*P_FBAin_*-*octt*-hisG-URA3-hiaG-Ku80dn cassette in PUC57 | This work |
| PUC57-IntE-*ACC1* | *ACC1* cassette in PUC57-IntE-HUM | This work |
| PUC57-IntE-*OLE1* | *OLE1* cassette in PUC57-IntE-HUM | This work |
| PUC57-K8FB-*OLE1* | *OLE1* cassette in PUC57-K8FB-HUM | This work |
| PMCS-URA | URA3 marker and guide RNA expression cassette in pMCSCen1 | [4] |
| CRI-PART2 | Cas9 expression cassette in PUC57 | [4] |
| PMCS-*PXA1* | Cas9 expression cassette in pMCSCen1 with *PXA1* gRNA | This work |
| PMCS-*PEX10* | Cas9 expression cassette in pMCSCen1 with *PEX10* gRNA | This work |
| PMCS-*MFE1* | Cas9 expression cassette in pMCSCen1 with *MFE1* gRNA | This work |
| PMCS-*POT1* | Cas9 expression cassette in pMCSCen1 with *POT1* gRNA | This work |
| PMCS-*RPD3* | Cas9 expression cassette in pMCSCen1 with *RPD3* gRNA | This work |
| PMCS-*SNF1* | Cas9 expression cassette in pMCSCen1 with *SNF1* gRNA | This work |
| PMCS-*LRO1* | Cas9 expression cassette in pMCSCen1 with *LRO1* gRNA | This work |
| PMCS-*PAH1* | Cas9 expression cassette in pMCSCen1 with *PAH1* gRNA | This work |
| PMCS-*DGK1* | Cas9 expression cassette in pMCSCen1 with *DGK1* gRNA | This work |
| PMCS-*MGA2* | Cas9 expression cassette in pMCSCen1 with *MGA2* gRNA | This work |
| PUC57-IntK-*αAS* | *αAS* cassette in PUC57-RS8d -HUM | This work |
| PUC57-IntK-*βAS* | *βAS* cassette in PUC57-RS8d -HUM | This work |
| PUC57-IntK-*TPS* | *TPS* cassette in PUC57-RS8d -HUM | This work |

**Table S7. Primer sequences** used in this work.

| Primer | Sequence | Source |
| --- | --- | --- |
| BsaI-*RcLus*-Hp8d-F | GGTCTCCAATGTGGCGAATCAAGATCGCC | This work |
| BsaI-*RcLus*-Hp8d-R | GGTCTCCTTTACAGGTTGTTGGTGAAGCCGAATC | This work |
| BsaI-*RcLus*-TEFin-F | GGTCTCCGCAGTGGCGAATCAAGATCGCCG | This work |
| BsaI-*RcLus*-TEFin-R | GGTCTCCTTTACAGGTTGTTGGTGAAGCCGAATC | This work |
| BsaI-*RcLus*-FBAin-F | GGTCTCCCCAGTGGCGAATCAAGATCGCCG | This work |
| BsaI-*RcLus*-FBAin-R | GGTCTCCTTTACAGGTTGTTGGTGAAGCCGAATC | This work |
| BsaI-PFBAin-*RcLus*–F | GGTCTCCCTGGGTTAGTTTGTGTAGAGAGTGTG | This work |
| BsaI-PFBAin-*RcLus*–R | GGTCTCCGCTTAACAGTGTACGCAGTACTATAGAGGAAC | This work |
| BsaI-*GFP*-Mit-F | GGTCTCCGCAGTTGTCTTTGAGACAATCTATCAGATTCTTCAAGAGATCTGGTATCCGAAAGGGTGAGGAGCTGTTCAC | This work |
| BsaI-*GFP*-Mit-R | GGTCTCCTTTACTTGTACAGCTCGTCC | This work |
| BsaI-*GFP*-Per-F | GGTCTCCGCAGCGAAAGGGTGAGGAGCTGTTCAC | This work |
| BsaI-*GFP*-Per-R | GGTCTCCTTTACAGCTTGGACTTGTACAGCTCGTCCATACCGTG | This work |
| BsaI- *GFP* -ER-F | GGTCTCCGCAGCGAAAGGGTGAGGAGCTGTTCAC | This work |
| BsaI- *GFP* -ER-R | GGTCTCCTTTACAGCTCGTCCTTCTTGTACAGCTCGTCCATACCGTG | This work |
| BsaI-*RcLus*-Mit-F | GGTCTCCGCAGTTGTCTTTGAGACAATCTATCAGATTCTTCAAGAGATCTGGTATCTGGCGAATCAAGATCGCCG | This work |
| BsaI-*RcLus*-Mit-R | GGTCTCCTTTACAGGTTGTTGGTGAAGCCGAATC | This work |
| BsaI-*RcLus*-Per-F | GGTCTCCGCAG GGCGAATCAAGATCGCCG | This work |
| BsaI-*RcLus*-Per-R | GGTCTCCTTTACAGCTTGGACAGGTTGTTGGTGAAGCCGAATC | This work |
| BsaI-*RcLus*-ER-F | GGTCTCCGCAGTGGCGAATCAAGATCGCCG | This work  This work  This work  This work  This work  This work |
| BsaI-*RcLus*-ER-R | GGTCTCCTTTACAGCTCGTCCTTCAGGTTGTTGGTGAAGCCGAATC |
| BsaI-IntE-*ACC1*-F | GGTCTCCGCAGATGAGATTGCAATTGAGAACTTTG |
| BsaI-IntE-*ACC1*-R | GGTCTCCTTTACAAACCCTTCAACAACTCAGC |
| BsaI-IntE-*OLE1*-F | GGTCTCCGCAGGCAGGTGAAGAACGTGGACCAG |
| BsaI-IntE-*OLE1*-R | GGTCTCCTTTATGGCGGCCATACCGGAC |
| BsaI-K8FB-*OLE1*-F | GGTCTCCCCAGGCAGGTGAAGAACGTGGACCAG | This work |
| BsaI-K8FB-*OLE1*-R | GGTCTCCTTTATGGCGGCCATACCGGAC | This work |
| *PXA1*-cas9-F | ACGTCTTCTCGTGGCCAAGCTCGA | This work |
| *PXA1*-cas9-R | AAACTCGAGCTTGGCCACGAGAAG | This work |
| *PEX10*-cas9-F | ACGTGCCCAGCCCGGAAACATGGA | This work |
| *PEX10*-cas9-R | AAACTCCATGTTTCCGGGCTGGGC | This work |
| *MFE1*-cas9-F | AAACCTGCTGGTCTTTACGGAAAC | This work |
| *MFE1*-cas9-R | ACGTGTTTCCGTAAAGACCAGCAG | This work |
| *POT1*-cas9-F | ACGTGGCCGCCGGCAAGTTCGACC | This work |
| *POT1*-cas9-R | AAACGGTCGAACTTGCCGGCGGCC | This work |
| *RPD3*-cas9-F | ACGTCCATCACGCAAAGAAATCCG | This work |
| *RPD3*-cas9-R | AAACCGGATTTCTTTGCGTGATGG | This work |
| *SNF1*-cas9-F | ACGTTTTGACTACATTGTGCAACG | This work |
| *SNF1-cas9-R* | AAACCGTTGCACAATGTAGTCAAA | This work |
| *LRO1-cas9-F* | AAACGACACAACCTGTGAATCGGA | This work |
| *LRO1-cas9-R* | ACGTTCCGATTCACAGGTTGTGTC | This work |

1. Hurt EC, Pesold-Hurt B, Suda K, Oppliger W, Schatz G. The first twelve amino acids (less than half of the pre-sequence) of an imported mitochondrial protein can direct mouse cytosolic dihydrofolate reductase into the yeast mitochondrial matrix. Embo J. 1985; 4(8): 2061-8.
2. Xu P, Qiao K, Ahn WS, Gregory S. Engineering *Yarrowia lipolytica* as a platform for synthesis of drop-in transportation fuels and oleochemicals. Procs Natl Acad Sci. 2016; 113(39): 10848-53.
3. Gao S, Tong Y, Zhu L, Ge M, Zhang Y, Chen D, et al. Iterative integration of multiple-copy pathway genes in *Yarrowia lipolytica* for heterologous beta-carotene production. Metab Eng. 2017; 41: 192-201.
4. Zhang JL, Peng YZ, Liu D, Liu H, Cao YX., Li BZ, et al. Gene repression via multiplex gRNA strategy in *Y. lipolytica*. Microb Cell Fact. 2018; 17(1): 62.
5. Jin CC, Zhang JL, Song H, Cao YX. Boosting the biosynthesis of betulinic acid and related triterpenoids in *Yarrowia lipolytica* via multimodular metabolic engineering. Microb Cell Fact. 2019; 18(1): 77.
