# Supplementary material for: High production of triterpenoids in Yarrowia lipolytica through manipulation of lipid components
Source: Biotechnol Biofuels. 2020 Jul 29;13:133. doi: 10.1186/s13068-020-01773-1 (PMC7392732; doi:10.1186/s13068-020-01773-1)
Supplement: Supplementary file 3 — Additional file 3: Methods. Culture optimization and cell integrity analysis. [file 13068_2020_1773_MOESM3_ESM.doc]

**Additional file 3.**

**Methods.**

**Culture optimization**

The glucose (Glu) in mYPD was replaced by glycerol (Gly) and citric acid (Cit) with equal carbon molar amounts. At the same time, attempts were made to combine these carbon sources with equal molar amounts (Glu-Gly, Glu-Cit and Gly-Cit). Unfortunately, the replacement and combination of other carbon sources did not improve the synthesis of lupeol in strain LU-23 (Additional file 1: Fig. S3A).

It has been reported that the pyruvate acid has positive effect on the improvement of terpenes production [1]. Therefore, the effect of gradient addition of pyruvate on the lupeol synthesis in strain LU-23 was explored. Optimized culture (containing 45.5 g/L glucose and 6 g/L pyruvate) were used and strains were cultured in 250 mL shake flasks for 120 h at 28 °C with shaking at 250 rpm, followed by inoculation to an optical density of 0.2 at 600 nm. The final strain LU-23 can produce 411.72 mg/L of lupeol in shake flask fermentation, which is a 32.20-fold increase compared to the original strain LU-6 (Additional file 1: Fig. S3B).

**Cell integrity analysis**

The propidium iodide staining method [2] was applied for cell integrity analysis. PI is a nuclear staining reagent that emits red fluorescence after embedding double-stranded DNA molecules (excitation: 535 nm; emission: 615 nm). As the experimental group, engineered cells of the fermentation broth were harvested after centrifugation and resuspended three times with ultrapure water. After centrifugation to remove the supernatant, the cells were resuspended with PI (concentration: 15 μM) phosphate buffer solution to an OD_600_ of 1, and protected from light for 15 min. As positive controls, control cells were resuspended with pure phosphate buffer solution to an OD600 of 1 (excluding PI), and protected from light for 15 min. 0.2 mL of cell suspensions were added into a 96-well plate (black plate, clear bottom). The relative fluorescence intensity of each sample was performed by using a microplate reader (SpectraMax M2, Molecular Devices, USA). And the PI absorption factor corresponding to each strain was calculated by the following formula:

1. Cao X, Lv YB, Chen J, Imanaka T, Wei LJ, Hua Q. Metabolic engineering of oleaginous yeast *Yarrowia lipolytica* for limonene overproduction. Biotechnol Biofuels. 2016; 9, 214.
2. Brennan TC, Turner CD, Kromer JO, Nielsen LK. Alleviating monoterpene toxicity using a two-phase extractive fermentation for the bioproduction of jet fuel mixtures in *Saccharomyces cerevisiae*. Biotechnol Bioeng. 2012; 109(10): 2513-22.
